# Efficacy of therapeutic interventions for idiopathic recurrent pregnancy loss: a systematic review and network meta-analysis

**DOI:** 10.3389/fmed.2025.1569819

**Published:** 2025-05-14

**Authors:** Jorge Lima, João Guerreiro, Miguel Ângelo-Dias, Sofia Silvério Serra, Teresa Costa, Natália Marto, João Feldman de Pinho, João Costa, Rodrigo Ruano, Gonçalo Silva Duarte

**Affiliations:** ^1^Department of Obstetrics and Gynecology, High-Risk Pregnancy Center, Hospital da Luz Lisboa, Lisbon, Portugal; ^2^Comprehensive Health Research Centre–CHRC, NOVA Medical School, Faculdade de Ciências Médicas, NMS, FCM, Universidade NOVA de Lisboa, Lisbon, Portugal; ^3^Laboratory of Clinical Pharmacology and Therapeutics, Faculdade de Medicina, Universidade de Lisboa, Lisbon, Portugal; ^4^Library, NOVA Medical School, Faculdade de Ciências Médicas, NMS, FCM, Universidade NOVA de Lisboa, Lisbon, Portugal; ^5^Department of Clinical Pharmacology, Hospital da Luz Lisboa, Lisbon, Portugal; ^6^Department of Reproductive Endocrinology and Infertility at the Reproductive Centers of America, New York, NY, United States; ^7^Division of Maternal-Fetal Medicine, Department of Obstetrics and Gynecology, University of Miami and Jackson Memorial Hospital, Miami, FL, United States; ^8^Clinical Pharmacology Unit, Unidade Local de Saúde Santa Maria, Lisbon, Portugal

**Keywords:** network meta-analysis, therapeutic interventions, idiopathic recurrent pregnancy loss, systematic review, live birth rate, miscarriage rate

## Abstract

**Background:**

Approximately 50% of cases of recurrent pregnancy loss (RPL) remain unexplained, and there is a lack of consensus concerning the effective treatments for idiopathic RPL. We used network meta-analyses to evaluate the efficacy of several prophylactic therapeutic interventions used in women with idiopathic RPL.

**Materials and methods:**

We conducted a systematic literature search using several databases from their inceptions to 20 July 2023. References from key articles were also manually searched. Randomized controlled trials assessing the efficacy and safety of any prophylactic intervention that were conducted in adult women with RPL were included. Studies with known causes of RPL were excluded. Two reviewers independently extracted data and assessed the risk of bias. Primary outcomes were live births and miscarriage rates. Secondary outcomes included serious adverse/adverse events and trial discontinuation. The network meta-analyses used a Bayesian hierarchical model with direct and indirect comparisons. Rank probabilities (assessed by surface under the cumulative ranking curve [SUCRA]) and certainty of evidence (assessed by Grading Recommendations Assessment, Development, and Evaluation [GRADE]) were also assessed.

**Results:**

Thirty-eight studies (6,379 participants) were evaluated. No statistically significant differences in live birth rates among the interventions were found. The three best-ranked interventions for this outcome were prednisone plus progesterone plus aspirin (83%), leukocyte immune therapy (74%), and prednisolone (65%). Women who were treated with progesterone plus human chorionic gonadotrophin (instead of a placebo) presented an increase in miscarriage odds (odds ratio [OR] 3.83, 95% credible intervals [CrIs] 1.04–14.38). The three best-ranked interventions for miscarriage rate were prednisone plus progesterone plus aspirin (SUCRA = 81%), hydroxychloroquine (SUCRA = 79%), and intralipid (SUCRA = 65%). Overall, under placebo, 59% (95% confidence interval [CI] 51–67; I^2^ = 92%) of participants underwent successful live births, and 35% (95% CI 30–42, I^2^ = 86%) underwent miscarriages. We found no evidence of statistically significant differences between interventions (the top three interventions were low-molecular-weight heparin, granulocyte colony-stimulating factor, and leukocyte immune therapy) in those who discontinued trial participation.

**Conclusion:**

Our results suggest that none of the analyzed interventions led to improvements in the live birth rate or a reduction in the miscarriage rate in women with idiopathic RPL.

**Systematic review registration:**

https://www.crd.york.ac.uk/prospero, identifier CRD42023455668.

## Introduction

Recurrent pregnancy loss (RPL) is defined as the failure of two or more clinical pregnancies before the point of fetal viability (up to 24 weeks of gestation). RPL presents a significant clinical challenge (1, 2). The prevalence of RPL has been reported as ranging from 0.8 to 3%, depending on population demographics, criteria for defining RPL, and the time of the study (3–8). The prevalence of RPL is nonetheless difficult to estimate due to challenges in obtaining accurate data concerning the number of experienced losses and the at-risk population of women, including all women of fertile age or those attempting to conceive. In addition, international guidelines vary in terms of their RPL definitions, with some of these guidelines defining it as two or more consecutive or non-consecutive pregnancy losses up to the 24th week of gestation (1, 2, 9), while others set the threshold at three or more losses up to the 14th (10) or 24th week (11). Therefore, such a lack of consensus can be challenging when comparing studies. However, clinicians are encouraged to use their clinical discretion to recommend extensive evaluation after two first-trimester miscarriages if the suspicion of a pathological nature of the losses is present.

Various causes and risk factors for RPL have been identified, including advanced maternal age, a history of multiple miscarriages, maternal distress, parental chromosomal abnormalities, uterine anatomical disorders, antiphospholipid syndrome, inherited thrombophilia, thyroid disorders, and environmental factors (12–16). However, approximately 50% of cases remain unexplained or idiopathic (1, 2, 11, 17). Such cases present a significant psychological burden for couples and healthcare providers (18). Since no evidence-based solutions for these women are available, treatment of these cases often involves empirical use of different treatment strategies, including acetylsalicylic acid, progesterone, corticosteroids, low-molecular-weight heparin (LMWH), intravenous immunoglobulin G (IVIG), lipid emulsion, and leukocyte immune therapy (19–21).

Despite thorough evaluations to identify presumptive risk factors and pathophysiologic mechanisms, physicians often fail to identify a specific target to direct a specific therapeutic intervention or prophylaxis for idiopathic RPL. Consequently, patients are often exposed to treatments based on theoretical hypotheses without proven efficacy (22).

Importantly, high-quality evidence regarding the therapeutic interventions of women with idiopathic RPL is scarce, and the current literature is insufficient to recommend any specific intervention for idiopathic RPL (1, 19). Furthermore, no systematic reviews and network meta-analyses (NMA) of randomized controlled trials (RCTs) have been published in which a comparison of the efficacy of the different therapeutic interventions used in women with idiopathic RPL may be particularly useful in assisting the clinical decision-making management options. Despite various proposed interventions, a lack of consensus exists concerning effective treatments for idiopathic RPL, thus emphasizing the need for this comprehensive network meta-analysis. Therefore, in this study, we used comprehensive NMA to evaluate the efficacy of various prophylactic therapeutic interventions for women with idiopathic RPL.

## Materials and methods

### Protocol and registration

This systematic review and NMA followed the Preferred Reporting Items Extension for Network Meta-Analyses (PRISMA-NMA; the checklist is presented in Supplementary Table S1) (23) guidelines and was registered with PROSPERO (ID: CRD42023455668) (24).

As a systematic review and NMA only involved the use of previously published data, no formal ethics approval or informed consent was required.

### Data sources

A systematic literature search was conducted using the PubMed, EMBASE, Cochrane Library, Scopus, and Web of Science databases from their inceptions to 20 July 2023. The search strategy included terms related to idiopathic recurrent pregnancy loss and therapeutic interventions (see Supplementary Table S2). References from the most relevant studies were hand-screened to identify any eventual missing publications not retrieved by the electronic search.

### Eligibility criteria and study selection

Inclusion criteria were RCTs that assessed the efficacy and safety of therapeutic interventions in adult women (>18 years) with idiopathic RPL.

RPL was defined as the loss of two or more clinical pregnancies before 24 weeks of gestation.

We excluded studies that included women with known diagnosed causes of RPL, including advanced maternal age (namely ≥40 years of age), parental chromosomal abnormalities, uterine anatomical disorders, inherited and/or acquired thrombophilia, thyroid disorders, and environmental factors. We also excluded cross-over trials due to the irrelevant nature of their study designs in the context of this review.

We imposed no restrictions on the number of recruited participants, number of recruitment centers, regional area, language, or year of publication. Unpublished studies (such as conference proceedings and poster or oral presentations) were also eligible for inclusion.

Two reviewers (GSD and JG) independently assessed all titles and abstracts of the retrieved search articles. Two reviewers (GSD and JG) conducted the selection of full-text articles for inclusion independently, and a third independent reviewer (JL) resolved any disagreements.

### Data collection process and data items

From each study meeting the inclusion criteria, two reviewers (GSD and JG) independently analyzed and collected information on study authors, year of publication, primary outcome of each RCT, median or mean age of participants, RPL definition, and treatment arms (therapeutic intervention and dose). Disagreements were resolved after discussion with a third reviewer (JL). When more information was needed, the corresponding authors of the included studies were contacted to obtain or confirm data.

### Primary and secondary outcome measures

Primary outcomes were the live birth and miscarriage rates. Secondary outcomes were serious adverse and adverse events and trial discontinuation.

We applied the International Council for Harmonization of Technical Requirements for Pharmaceuticals for Human Use (ICH) definitions of serious adverse events and adverse events (25).

### Assessment of risk of bias within individual studies

Two reviewers (GSD and JG) independently evaluated the trial-level risk of bias using the Cochrane risk of bias tool for randomized trials (26). Disagreements were resolved after a discussion with a third reviewer (JC). Each trial’s overall risk of bias was divided into high or low risk.

### Statistical analyses

The NMA involved both direct and indirect comparisons and was performed using a Bayesian hierarchical model (binomial modeling with a rate logit link function) supplemented with a Markov chain Monte Carlo approach (27, 28). We performed 10,000 adaptation steps followed by 100,000 iterations with a thinning factor of 10. All potential scale reduction factors were less than 1.05, indicating good convergence.

We constructed a network diagram for each outcome to illustrate all comparisons between therapeutic interventions. Each intervention was represented as a separate node, and the comparisons between interventions were depicted as links connecting these nodes. Node sizes corresponded to the number of participants who received an intervention, and connection sizes corresponded to the number of trials within a given comparison. Different doses of the same intervention were clustered into a single node.

For all models, we used vague prior distributions for all trial baselines and relative treatment or class effects and specifically selected normal distributions with a mean of 0 and a variance of 100^2^. For the random treatment effects models, we applied a minimally informative uniform prior distribution for the between-study heterogeneity parameter. For exchangeable-class models, we used a uniform (0, 5) prior distribution for the within-class standard deviation.

Fixed-, random-, and unrelated mean effects models were applied to each outcome and compared regarding the total residual deviances and the total number of data points to select the model that best fits the data. All outcomes were analyzed as binary variables using log odds ratios (log ORs), a binomial likelihood, and a complementary log–log (cloglog) link function. The outcomes were reported as odds ratios (ORs) with 95% confidence intervals (CIs) or 95% credible intervals (CrIs) as applicable. Heterogeneity between the included studies was evaluated using the heterogeneity index (I^2^) statistic and τ^2^ (tau-squared) value. A τ^2^ value greater than 0.5% indicated high statistical heterogeneity. Potential inconsistencies between direct and indirect evidence were assessed using the node-splitting method. To rank the therapeutic interventions, we used the surface under the cumulative ranking curve (SUCRA), for which higher values indicate a higher probability that a given intervention is associated with a better outcome. Publication bias was assessed using Peter’s test. Statistical significance was considered as *p* < 0.05 for all analyses. All statistical analyses were performed in R (version 4.3.2, R Foundation for Statistical Computing, Vienna, Austria) using the ‘gemtc’ package (version 0.8–7, GitHub, Inc., San Francisco, CA).

### Assessment of certainty of evidence

The Grading of Recommendations Assessments, Development, and Evaluation (GRADE) approach was used to assess the certainty of the evidence for the outcomes of live birth and miscarriage rates and trial discontinuation of this NMA (29). Judgments concerning the certainty of evidence were obtained for several domains: (1) risk bias within studies, (2) indirectness, (3) inconsistency, and (4) imprecision. Supplementary Tables S3–S5 list all details of the GRADE assessment for each outcome.

## Results

### Study selection

**Figure 1:**
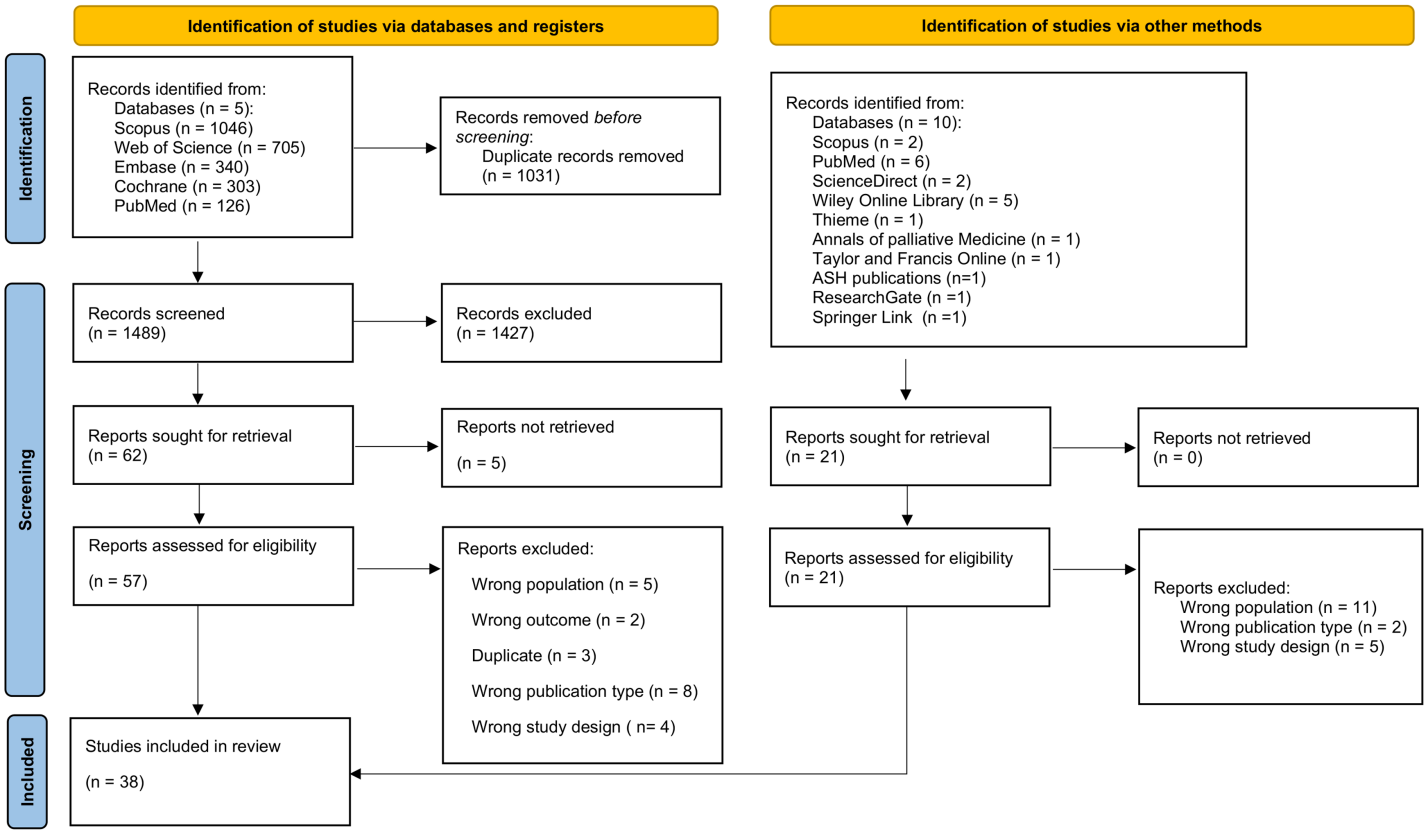
Preferred reporting items for systemic reviews and meta-analyses (PRISMA) 2020 flow diagram of systematic review process and study selection.

Figure 1 depicts the PRISMA 2020 flow diagram for the systematic review process and study selection.

Our database search yielded 2,520 records, and after manually searching reference lists, we found an additional 21 records. We excluded 1,031 duplicates and 1,427 other records based on title and abstract screening. Five records were not retrieved, and after the review of 57 full-text articles, 22 studies were excluded due to wrong sample population (*n* = 5), wrong outcome (*n* = 2), duplicate (*n* = 3), wrong publication type (*n* = 8), and wrong study design (*n* = 4). In total, 38 RCTs (30–67) fulfilled all inclusion criteria and were included in our systematic review (Figure 1).

### General characteristics of the studies

A total of 38 RCTs (30–67) with 6,379 participants were included in this study. Study characteristics are detailed in Table 1. The included studies were published between 1993 and 2022, and all were available in full-text format.

**Table 1:** Characteristics of the included studies.

References	Primary outcome	N randomized	RPL definition	Age (median/mean)	Moment when interventions were initiated	Arm 1 (dose)	Arm 2 (dose)	Arm 3 (dose)
Akbari et al. (50)	Live birth rate	*N* = 173 Arm 1: *n* = 85 Arm 2: n = 88	≥2	29.9	Since positive pregnancy test (aspirin and LMWH) until week 32 (aspirin) and discontinued 24–48 h before delivery (LMWH)	Aspirin (80 mg oral) daily plus LMWH (enoxaparin 40 mg daily till week 36 and after heparin sodium 5,000 UI SC) twice daily	Aspirin (80 mg oral) daily	
Blomqvist et al. (30)	Live birth rate	*N* = 400 Arm 1: *n* = 200 Arm 2: n = 200	≥3	32.3	Since positive pregnancy test until week 36	Aspirin (75 mg oral) daily	Placebo	
Chen et al. (31)	Pregnancy for > 20 weeks	*N* = 749 Arm 1: *n* = 380 Arm 2: *n* = 369	≥2	28.5	Four treatments before gestation (every 2–3 weeks) and three after pregnancy	Leukocyte immune therapy 0.2 mL SC every 2–3 weeks	Progesterone (dose NS)	
Christiansen et al. (32)	Live birth rate	*N* = 82 Arm 1: *n* = 42 Arm 2: *n* = 40	≥4	32.4	Since positive pregnancy test a total of eight infusions were given until gestational week 15	IVIG If < 75 kg 24 g (200 mL) and if >75 kg 36 g (300 mL)	Placebo (200 or 300 mL 5% albumin)	
Christiansen et al. (33)	Healthy pregnancy at 28 weeks of gestation	*N* = 34 Arm 1: *n* = 17 Arm 2: *n* = 17	≥3	NS	Since positive pregnancy test until gestational week 34	IVIG doses were adjusted according to weight, varying infusion doses between 465 and 550 g	Placebo	
Christiansen et al. (34)	Miscarriage rate	*N* = 66 Arm 1: *n* = 43 Arm 2: *n* = 23	≥2	29.6	Preconception. Repeated treatment every month until conception	Leukocyte immune therapy (150 mL autologous blood IV every 5 months)	Placebo	
Coomarasamy et al. (35)	Live birth after 24 weeks of gestation	*N* = 836 Arm 1: *n* = 404 Arm 2: *n* = 423	≥3	32.7	Since positive pregnancy test until gestational week 12	Progesterone (400 mg vaginal) twice daily	Placebo	
Coulam et al. (36)	Live birth rate	*N* = 95 Arm 1: *n* = 47 Arm 2: *n* = 48	≥2	35.0	Preconception. Every 28 days until pregnancy or for 4 months	IVIG (500 mg/Kg IV) month	Placebo	
Dolitzky et al. (37)	Live birth rate or miscarriage rate	*N* = 104 Arm 1: *n* = 54 Arm 2: *n* = 55	≥3	31.19	Since fetal heartbeat detected until gestational week 37	LMWH (enoxaparin 40 mg SC) daily	Aspirin (100 mg oral) daily	
Eapen et al. (38)	Clinical pregnancy at 20 weeks of gestation	*N* = 150 Arm 1: *n* = 76 Arm 2: *n* = 74	≥3	31.5	Since positive pregnancy test until gestational week 9	G-CSF (130 μg SC) daily	Placebo	
El-Zibdeh (39)	Miscarriage rate/ Live birth rate	*N* = 180 Arm 1: *n* = 82 Arm 2: *n* = 50 Arm 3: *n* = 48	≥3	NS	Since positive pregnancy test until gestational week 12	Dydrogesterone (10 mg oral) twice daily	hCG (5,000 IU IM) every 4 days	Placebo
Elmahashi et al. (40)	Live birth rate or miscarriage rate	*N* = 150 Arm 1: *n* = 75 Arm 2: *n* = 75	≥3	26.9	Since fetal heartbeat detected until gestational week 34	Aspirin (75 mg oral) daily	Aspirin (75 mg oral) plus LMWH (0.4 mL SC) daily	
Fawzy et al. (41)	Live birth rate	*N* = 170 Arm 1: *n* = 57 Arm 2: *n* = 53 Arm 3: *n* = 50	≥3	21.8	Since pregnancy until gestational week 12 (prednisone and progesterone) and week 32 (aspirin)	LMWH (enoxaparin 20 mg SC) daily	Prednisone (20 mg oral) plus progesterone (20 mg oral) plus aspirin (75 mg oral) daily	Placebo
Gatenby et al. (42)	Live birth rate	*N* = 41 Arm 1: *n* = 19 Arm 2: *n* = 22	≥3	32.8	Preconception	Leukocyte immune therapy (400 × 10^6^ PBML were suspended in 5 mL medium. Three ml of the cell suspension was given IV and 0.5 mL into each of two intradermal and two subcutaneous sites on the forearm) once	Placebo	
Ghosh et al. (43)	Endometrial blood flow parameters by Doppler indices and ongoing pregnancy rate	*N* = 101 Arm 1: *n* = 50 Arm 2: *n* = 51	≥3	28.8	Since positive pregnancy test until gestational week 12	Dydrogesterone (10 mg oral) twice daily	Progesterone (100 mg vaginal) thrice daily	
Gomaa et al. (44)	Live birth rate	*N* = 160 Arm 1: *n* = 80 Arm 2: *n* = 80	≥2	26.6	Since viable current early pregnancy (< 7 gestational weeks)	Prednisolone (5 mg oral) daily	Placebo	
Jablonowska et al. (45)	Live birth rate	*N* = 41 Arm 1: *n* = 22 Arm 2: *n* = 19	≥3	30.0	Every 3 weeks on five occasions if a viable pregnancy was confirmed by ultrasound before each treatment	IVIG (20 g, 400 mL IV)	Placebo	
Kaandorp et al. (46)	Live birth rate	*N* = 364 Arm 1: *n* = 123 Arm 2: *n* = 120 Arm 3: *n* = 121	≥2	34.0	Preconception or at a gestational age of less than 6 weeks and up to week 36 (aspirin and placebo) Since a viable intrauterine pregnancy until labor (LMWH)	Aspirin (100 mg oral) plus LMWH (2,850 UI SC) daily	Aspirin (100 mg oral) daily	Placebo
Khan et al. (47)	Live birth rate	*N* = 80 Arm 1: *n* = 80 Arm 2: *n* = 80	≥2	26.0	Since fetal heartbeat detected until delivery	LMWH (enoxaparin 40 mg SC) daily	Placebo	
Li et al. (48)	Live birth rate	*N* = 124 Arm 1: *n* = 62 Arm 2: *n* = 62	≥2	27.3	Preconception (leukocyte immune therapy). After pregnancy confirmation and continued for 3 months (Progesterone and hCG).	Leukocyte immune therapy (2–4 × 10^7^/ml SC) once	Progesterone (100 mg oral) daily for 14 days hCG (2000 U IM) twice daily	
Meng et al. (49)	Rate of successful pregnancy	*N* = 192 Arm 1: *n* = 96 Arm 2: *n* = 96	≥3	31.4	Since preconception until gestational week 12	Intralipid (20% 250 mL IV) every 2 weeks before pregnancy and once a week after pregnancy confirmation	IVIG (25 g IV) every 4 weeks before pregnancy and once a week after pregnancy confirmation	
Moini et al. (51)	Incidence of abortion	*N* = 29 Arm 1: *n* = 14 Arm 2: *n* = 15	≥2	30.9	Since pregnancy positive test until gestational week 20	Hydroxychloroquine (200 mg oral) twice daily	Placebo	
Nazari et al. (52)	Live birth rate	*N* = 60 Arm 1: *n* = 28 Arm 2: *n* = 32	≥3	30.5	Since positive pregnancy test until gestational week 24 (IVIG) or week 37 (LMWH and aspirin)	IVIG (200 mg/kg IV) monthly plus LMWH (enoxaparin 40 mg SC) daily plus aspirin (80 mg oral) daily	LMWH (enoxaparin 40 mg SC) daily plus aspirin (80 mg oral) daily	
Ober et al. (53)	Live birth rate	*N* = 183 Arm 1: *n* = 91 Arm 2: *n* = 92	≥3	32.7	Preconception	Leukocyte immune therapy (3 mL IV + 0–5 mL [2x] intradermic)	Placebo	
Pasquier et al. (54)	Live birth rate	*N* = 258 Arm 1: *n* = 138 Arm 2: *n* = 120	≥2	32.4	Since pregnancy positive test until gestational week 35.	LMWH (enoxaparin 40 mg SC) daily	Placebo	
Perino et al. (55)	Live birth rate or miscarriage rate	*N* = 46 Arm 1: *n* = 22 Arm 2: *n* = 24	≥3	29.7	Following a positive pregnancy test, patients received two initial doses on 2 consecutive days and a third dose 3 weeks later when ultrasound confirmed an ongoing pregnancy.	IVIG (two initial doses of 25 g/day IV) on 2 consecutive days and a third dose of 25 g 3 weeks later	Placebo	
Quenby et al. (56)	Live birth rate	*N* = 81 Arm 1: *n* = 42 Arm 2: *n* = 39	≥2	29.4	Since pregnancy positive test until gestational week 14	hCG (10.000 UI and then 5.000 UI IM) twice a week	Placebo	
The German RSA/IVIG Group (63)	Live birth rate	*N* = 64 Arm 1: *n* = 33 Arm 2: *n* = 31	≥3	28.5	Since pregnancy positive test until gestational week 25	IVIG (600 mL first dose, next doses 400 mL IV) every 3 weeks	Placebo	
Scarpellini et al. (57)	Live birth rate	*N* = 68 Arm 1: *n* = 35 Arm 2: *n* = 33	≥4	34.4	From the sixth day after ovulation until the occurrence of menstruation or to the end of gestation week 9	G-CSF (1 μg/Kg/day SC)	Placebo	
Schleussner et al. (58)	Pregnancy at 24 weeks of gestation	*N* = 449 Arm 1: *n* = 226 Arm 2: *n* = 223	≥2	32.1	Since fetal heartbeat detected until gestational week 24.	LMWH (dalteparin–sodium 5,000 IU SC) daily plus multivitamins containing folic acid daily	Multivitamins containing folic acid daily	
Shaaban et al. (59)	Clinical pregnancy at 20 weeks of gestation	*N* = 300 Arm 1: *n* = 150 Arm 2: *n* = 150	≥3	26.6	Positive pregnancy test until gestational week 20	LMWH (tinzaparin 4,500 IU SC) daily plus folic acid (500 μg oral) daily	Folic acid (500 μg Oral) daily	
Stephenson et al. (60)	Clinical pregnancy at 20 weeks of gestation	*N* = 77 Arm 1: *n* = 38 Arm 2: *n* = 39	≥3	35.5	Preconception and during pregnancy every 4 weeks until gestational week 18–20	IVIG (500 mg/Kg)	Placebo	
Stephenson et al. (61)	Clinical pregnancy at 20 weeks of gestation	*N* = 60 Arm 1: *n* = 32 Arm 2: *n* = 30	≥2	31.4	Preconception (follicular phase for a maximum of six menstrual cycles)	IVIG (500 mg/Kg)	Placebo	
Tang et al. (62)	Live birth rate	*N* = 40 Arm 1: *n* = 20 Arm 2: *n* = 20	≥3	33.5	Since fetal heartbeat detected until gestational week 12	Prednisolone (oral) 20 mg daily for 6 weeks, 10 mg daily for 1 week and then 5 mg daily for 1 week	Placebo	
Xu et al. (64)	Live birth rate	*N* = 120 Arm 1: *n* = 60 Arm 2: *n* = 60	≥3	30.6	Since pregnancy until gestational week 12	LMWH (dalteparin–sodium 5,000 IU SC) daily	Progesterone (20 mg IM) daily plus hCG (dose NS) daily	
Yamada et al. (65)	Clinical pregnancy at 22 weeks of gestation	*N* = 102 Arm 1: *n* = 53 Arm 2: *n* = 49	≥4	35.1	Treatment initiated at 4 to 6 weeks gestation and for 5 consecutive days	IVIG (400 mg/Kg) daily	Placebo	
Zafardoust et al. (66)	Clinical pregnancy or miscarriage rate	*N* = 50 Arm 1: *n* = 23 Arm 2: *n* = 27	≥3	30.9	Preconception (G-CSF) Since pregnancy (aspirin and LMWH) until gestational week 20	G-CSF (300 μg intrauterine injection) twice in the cycle plus aspirin (80 mg oral) daily plus LMWH (5,000 U SC) daily	Aspirin (80 mg Oral) daily plus LMWH (5,000 U SC) daily	
Zolghadri et al. (67)	Live birth rate	*N* = 100 Arm 1: *n* = 50 Arm 2: *n* = 50	≥3	36.4	Since fetal heart beat detected until gestational week 36	LMWH (dalteparin–sodium 5,000 U SC) twice a day plus aspirin (80 mg oral) daily	Placebo	

G-CSF, granulocyte colony-stimulating factor; hCG, human chorionic gonadotropin; IM, intramuscular; IV, intravenous; IVIG, intravenous immunoglobulin G; LMWH, low-molecular-weight heparin; NS, Not specified; PBML, peripheral blood mononuclear leukocyte; RPL, recurrent pregnancy loss; SC subcutaneous.

Among these 38 articles, 13 (31, 34, 36, 44, 46–48, 50, 51, 54, 56, 58, 61) defined RPL as two or more miscarriages, 22 (30, 33, 35, 37–43, 45, 49, 52, 53, 55, 59, 60, 62–64, 66, 67) as three or more miscarriages, and three (32, 57, 65) as four or more miscarriages (Table 1).

Overall, the included studies included the following active interventions: (1) aspirin; (2) aspirin plus LMWH; (3) granulocyte colony-stimulating factor (G-CSF); (4) G-CSF plus aspirin plus LMWH; (5) human chorionic gonadotropin (hCG); (6) hydroxychloroquine; (7) intralipid; (8) IVIG; (9) IVIG plus LMWH plus aspirin; (10) leukocyte immune therapy; (11) LMWH; (12) prednisolone; (13) prednisone plus progesterone plus aspirin; (14) progesterone; (15) progesterone plus hCG (Table 1).

Participant ages ranged from 21.8 to 36.4 years on average. RCTs were generally two-arm trials (*n* = 35) (30–38, 40, 42–45, 47–67), with a smaller number being three-arm trials (n = 3) (39, 41, 46). The two most frequently studied therapeutic interventions were IVIG (*n* = 9) (32, 33, 36, 45, 55, 60, 61, 63, 65) and LMWH (*n* = 5) (37, 41, 47, 54, 64) (Table 1).

RCTs were not found to assess the efficacy and safety of levothyroxine, folic acid, multivitamins, clomiphene citrate, sitagliptin, metformin, and vitamin D for RPL (Table 1).

### Risk of bias within individual studies

From the 38 included RCTs, 16 (41%) (31, 39, 40, 45–50, 52, 53, 58–60, 64, 67) were rated as having a high risk of bias. The main domains contributing to the bias rating were the risk of performance and detection bias. However, we also assessed six trials at a high (45, 49, 60) or unclear (46, 53, 59) risk of attrition bias (Table 2).

**Table 2:** Risk of bias assessment within individual studies according to the Cochrane risk of bias tool for randomized trials.

**Study**	**Random sequence generation**	**Allocation concealment**	**Performance**	**Detection**	**Attrition**	**Selective reporting**	**Other**	**Overall bias**
Akbari et al. (50)	Low	Low	High	High	Low	Low	Low	High
Blomqvist et al. (30)	Low	Low	Low	Low	Low	Low	Low	Low
Chen et al. (31)	Low	Low	Unclear	Unclear	Low	Low	Low	High
Christiansen et al. (32)	Low	Low	Low	Low	Low	Low	Low	Low
Christiansen et al. (33)	Low	Low	Low	Low	Low	Low	Low	Low
Christiansen et al. (34)	Low	Low	Low	Low	Low	Low	Low	Low
Coomarasamy et al. (35)	Low	Low	Low	Low	Low	Low	Low	Low
Coulam et al. (36)	Low	Low	Low	Low	Low	Low	Low	Low
Dolitzky et al. (37)	Low	Low	Low	Low	Low	Low	Low	Low
Eapen et al. (38)	Low	Low	Low	Low	Low	Low	Low	Low
El-Zibdeh (39)	Low	Low	Unclear	Unclear	Low	Low	Low	High
Elmahashi et al. (40)	Low	Low	Unclear	Unclear	Low	Low	Low	High
Fawzy et al. (41)	Low	Low	Low	Low	Low	Low	Low	Low
Gatenby et al. (42)	Low	Low	Low	Low	Low	Low	Low	Low
Ghosh et al. (43)	Low	Low	Low	Low	Low	Low	Low	Low
Gomaa et al. (44)	Low	Low	Low	Low	Low	Low	Low	Low
Jablonowska et al. (45)	Low	Low	Low	Low	High	Low	Low	High
Kaandorp et al. (46)	Low	Low	High	High	Unclear	Low	Low	High
Khan et al. (47)	Low	Low	Unclear	Unclear	Low	Low	Low	High
Li et al. (48)	Low	Low	Unclear	Unclear	Low	Low	Low	High
Meng et al. (49)	Low	Low	Unclear	Unclear	High	Low	Low	High
Moini et al. (51)	Low	Low	Low	Low	Low	Low	Low	Low
Nazari et al. (52)	Low	Low	Unclear	Unclear	Low	Low	Low	High
Ober et al. (53)	Low	Low	Low	Low	Unclear	Low	Low	High
Pasquier et al. (54)	Low	Low	Low	Low	Low	Low	Low	Low
Perino et al. (55)	Low	Low	Low	Low	Low	Low	Low	Low
Quenby and Farquharson (56)	Low	Low	Low	Low	Low	Low	Low	Low
The German RSA/IVIG Group (63)	Low	Low	Low	Low	Low	Low	Low	Low
Scarpellini and Sbracia (57)	Low	Low	Low	Low	Low	Low	Low	Low
Schleussner et al. (58)	Low	Low	High	High	Low	Low	Low	High
Shaaban et al. (59)	Low	Low	Low	Low	Unclear	Low	Low	High
Stephenson et al. (60)	Low	Low	Low	Low	High	Low	Low	High
Stephenson et al. (61)	Low	Low	Low	Low	Low	Low	Low	Low
Tang et al. (62)	Low	Low	Low	Low	Low	Low	Low	Low
Xu et al. (64)	Low	Low	Unclear	Unclear	Low	Low	Low	High
Yamada et al. (65)	Low	Low	Low	Low	Low	Low	Low	Low
Zafardoust et al. (66)	Low	Low	Low	Low	Low	Low	Low	Low
Zolghadri et al. (67)	Low	Low	Unclear	Unclear	Low	Low	Low	High

### Model properties

Overall, we applied an NMA for the three outcomes: (1) live birth rate, (2) miscarriage rate, and (3) trial discontinuation. Despite being unable to conduct NMA due to a lack of data regarding both serious adverse and adverse events, we present the available data regarding these outcomes in Supplementary Tables S6, S7, respectively.

Supplementary Tables S8–S10 detail the model fit for each outcome. For all outcomes, only the random-effect models had similar total residual deviances when compared with the total number of data points, indicating an adequate fit of the results (Supplementary Tables S8–S10). Therefore, the results presented throughout this review pertain exclusively to the random-effects models.

**Figure 2:**
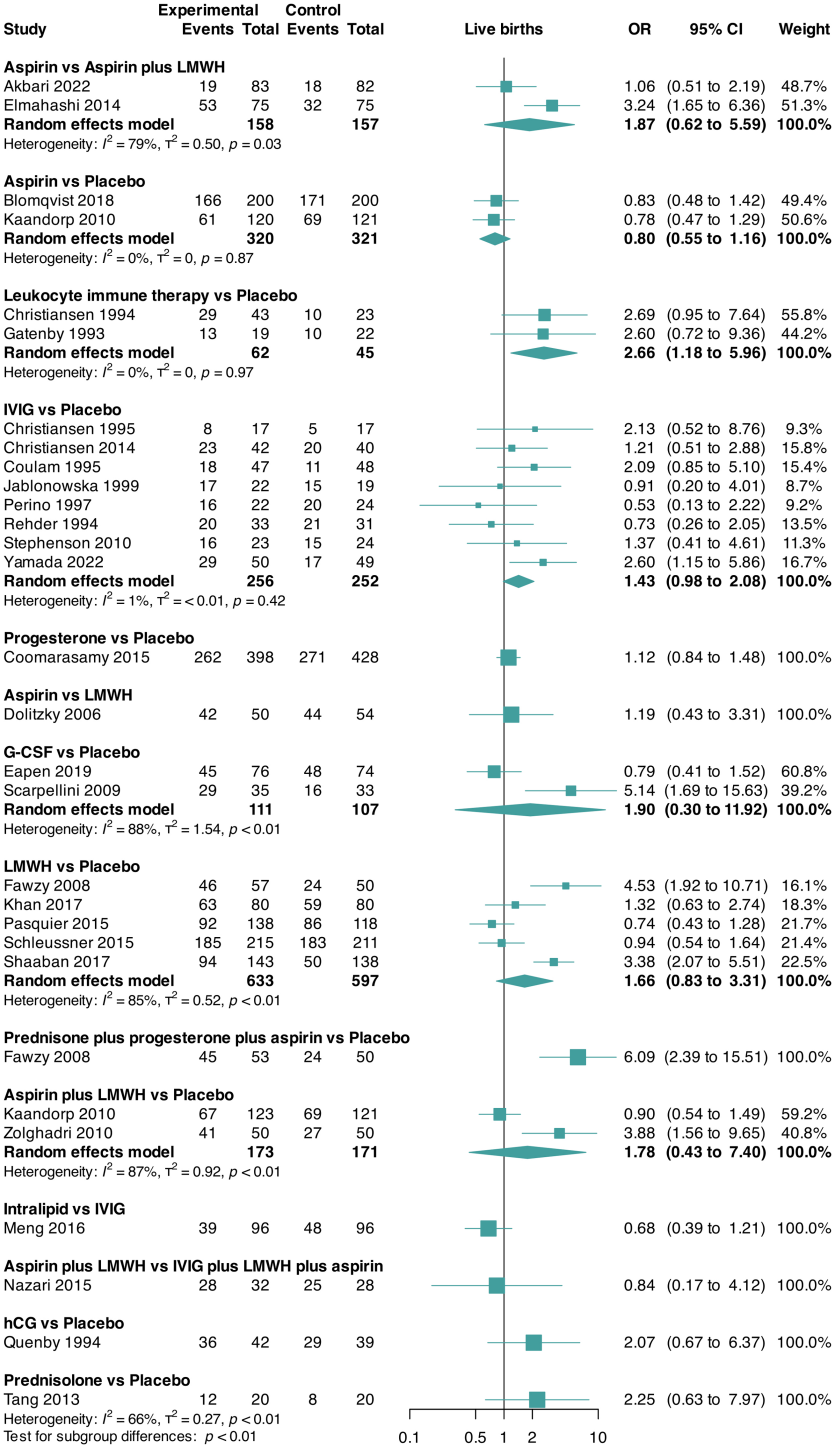
Pairwise meta-analysis for the outcome live birth rate. CI, confidence interval; G-CSF, granulocyte colony-stimulating factor; hCG, human chorionic gonadotropin; IVIG, intravenous immunoglobulin G; LMWH, low-molecular-weight heparin; OR, odds ratio.

**Figure 3:**
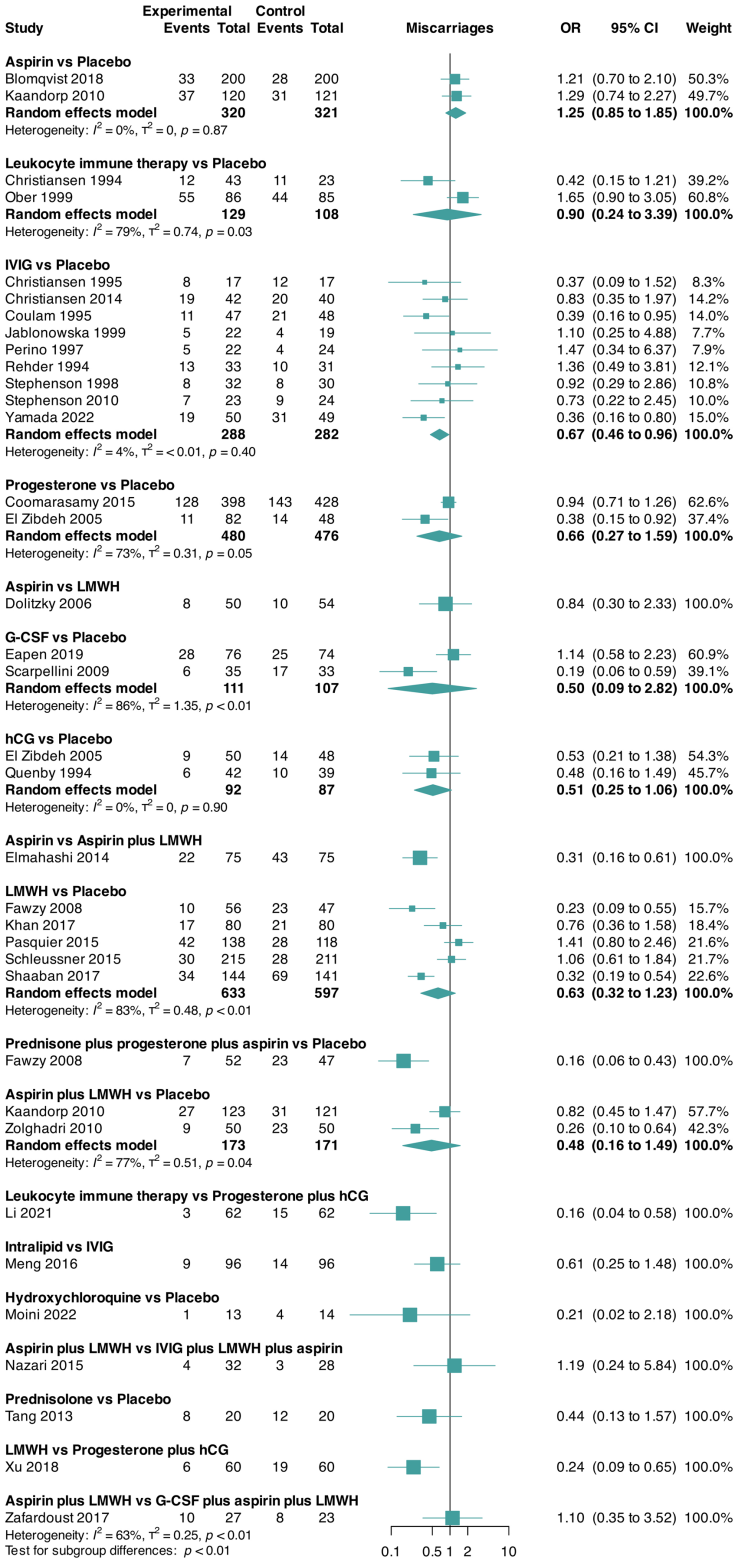
Pairwise meta-analysis for the outcome miscarriage rate. CI, confidence interval; G-CSF, granulocyte colony-stimulating factor; hCG, human chorionic gonadotropin; IVIG, intravenous immunoglobulin G; LMWH, low-molecular-weight heparin; OR, odds ratio.

**Figure 4:**
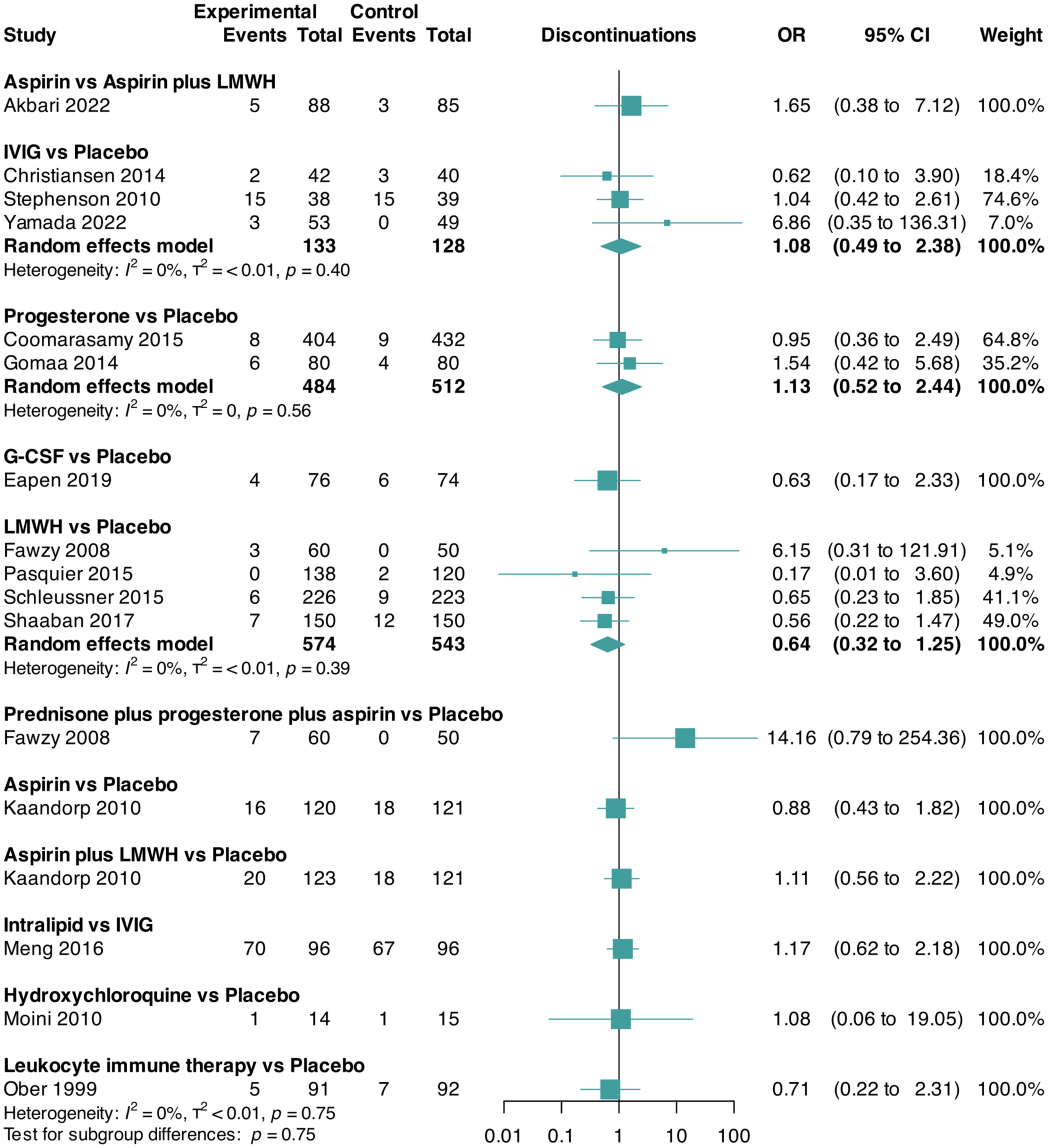
Pairwise meta-analysis for the outcome trial discontinuation. CI, confidence interval; G-CSF, granulocyte colony-stimulating factor; IVIG, intravenous immunoglobulin G; LMWH, low-molecular-weight heparin; OR, odds ratio.

The pairwise meta-analyses for live birth and miscarriage rates and trial discontinuation are presented in Figures 2–4, respectively. Regarding heterogeneity in pairwise meta-analysis, we found moderate-to-high levels of heterogeneity as reflected by high levels of tau^2^ and/or *I*^2^ in four of 14 contrasts in the NMA of live birth rate (aspirin versus aspirin plus LMWH [two trials], G-CSF versus placebo [two trials], LMWH versus placebo [five trials], and aspirin plus LMWH versus placebo [two trials]). In the miscarriage rate NMA, we found moderate-to-high levels of heterogeneity in five of 18 contrasts (leukocyte immune therapy versus placebo [two trials], progesterone versus placebo [two trials], G-CSF versus placebo [two trials], LMWH versus placebo [five trials], and aspirin plus LMWH versus placebo [two trials]). No contrasts in the NMA of trial discontinuations were found. Moreover, regarding heterogeneity, the between-study standard deviations across all outcomes were considered acceptable (Figures 2–4). Regarding inconsistency, the parameter estimates were similar for both the random effects and unrelated mean effects models, and considerable overlap in the 95% CrIs was observed (Supplementary Tables S8–S10). This finding suggests no evidence of global inconsistency in the network.

Regarding inconsistency between direct and indirect evidence, node-split models suggest inconsistency in the comparison of placebo versus aspirin in both the outcomes live birth rate (Supplementary Figure S1) and miscarriage rate (Supplementary Figures S2, S3). Node-split analysis was not possible for the trial discontinuation outcome because of the network geometry for this outcome.

### Live birth rate

**Figure 5:**
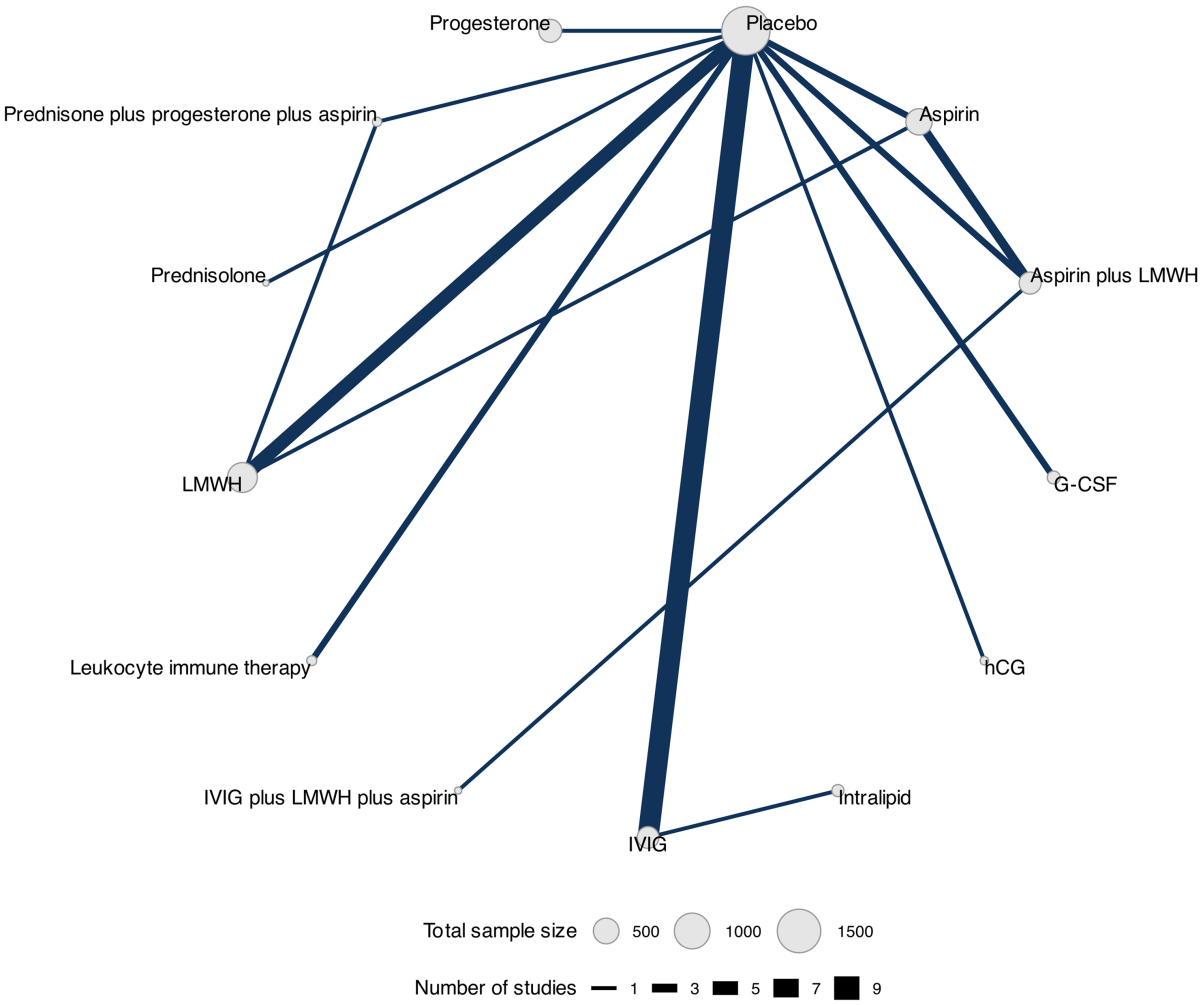
Network plot for live birth rate. Network plot showing comparisons in live birth rate between nodes (gray circles) in which each node represents a therapeutic intervention. The size of each node is proportional to the total number of participants assigned to that intervention, while the width of each connecting line is proportional to the number of studies conducting head-to-head comparisons between the two nodes.

The network plot with the comparisons between the therapeutic interventions for live birth rate is shown in Figure 5.

Data for this outcome were reported in 28 RCTs (30, 32–38, 40–42, 45–47, 49, 50, 52, 54–60, 62, 63, 65, 67) (4,598 participants) that compared 13 interventions. Two RCTs were three-arm trials (41, 46) (Figure 2).

The network meta-analysis showed no statistically significant differences in live birth rates among the interventions (Table 3 and Supplementary Figure S4). The best-ranked interventions (according to the SUCRA, where higher values indicate more certainty that the intervention is the best-ranked in the comparison), as shown in Table 4, were prednisone plus progesterone plus aspirin (SUCRA = 83%), leukocyte immune therapy (SUCRA = 74%), and prednisolone (SUCRA = 65%). We did not find evidence of publication bias for this outcome (Peters test, *p* = 0.088), and the certainty of evidence of the relative treatment effects varied from low to moderate (Supplementary Table S3).

**Table 3:** League table showing the comparisons for the efficacy of each therapeutic intervention for live birth rate.

Aspirin	0.87 (0.42 to 1.82)	1.24 (0.35 to 4.68)	1.49 (0.25 to 9.12)	0.68 (0.13 to 3.4)	0.99 (0.38 to 2.49)	1.09 (0.13 to 10.06)	1.95 (0.48 to 8)	1.15 (0.47 to 2.72)	0.73 (0.34 to 1.5)	1.67 (0.25 to 11.17)	2.72 (0.57 to 12.89)	0.82 (0.19 to 3.43)
1.15 (0.55 to 2.36)	Aspirin plus LMWH	1.42 (0.38 to 5.52)	1.71 (0.27 to 10.9)	0.79 (0.14 to 3.92)	1.13 (0.4 to 3)	1.25 (0.17 to 9.94)	2.24 (0.53 to 9.54)	1.32 (0.5 to 3.39)	0.84 (0.36 to 1.87)	1.91 (0.28 to 13.29)	3.11 (0.62 to 15.26)	0.93 (0.2 to 4.11)
0.81 (0.21 to 2.9)	0.71 (0.18 to 2.62)	G-CSF	1.21 (0.17 to 8.44)	0.55 (0.09 to 3.13)	0.8 (0.23 to 2.52)	0.88 (0.08 to 10.2)	1.57 (0.31 to 7.58)	0.93 (0.27 to 3)	0.59 (0.2 to 1.64)	1.34 (0.18 to 10.49)	2.2 (0.37 to 12.3)	0.66 (0.12 to 3.25)
0.67 (0.11 to 4.03)	0.58 (0.09 to 3.74)	0.83 (0.12 to 5.91)	hCG	0.45 (0.05 to 4)	0.66 (0.11 to 3.7)	0.73 (0.05 to 11.76)	1.31 (0.17 to 9.8)	0.77 (0.13 to 4.41)	0.49 (0.09 to 2.55)	1.12 (0.1 to 12.27)	1.8 (0.21 to 16.3)	0.54 (0.07 to 4.31)
1.47 (0.29 to 7.74)	1.27 (0.25 to 6.95)	1.82 (0.32 to 11.49)	2.22 (0.25 to 20.68)	Intralipid	1.44 (0.38 to 5.51)	1.62 (0.12 to 23.73)	2.91 (0.45 to 19.22)	1.68 (0.36 to 8.08)	1.07 (0.25 to 4.66)	2.46 (0.26 to 24.88)	4 (0.55 to 30.65)	1.2 (0.18 to 8.2)
1.01 (0.40 to 2.65)	0.88 (0.33 to 2.51)	1.26 (0.40 to 4.39)	1.52 (0.27 to 8.95)	0.69 (0.18 to 2.63)	IVIG	1.1 (0.12 to 11.27)	1.97 (0.53 to 7.61)	1.16 (0.52 to 2.71)	0.74 (0.41 to 1.34)	1.7 (0.27 to 10.92)	2.74 (0.62 to 12.69)	0.83 (0.21 to 3.32)
0.92 (0.10 to 7.79)	0.80 (0.10 to 6.01)	1.14 (0.1 to 12.83)	1.36 (0.09 to 21.19)	0.62 (0.04 to 8.41)	0.9 (0.09 to 8.62)	IVIG plus LMWH plus aspirin	1.79 (0.14 to 20.74)	1.05 (0.1 to 9.74)	0.67 (0.07 to 5.8)	1.54 (0.09 to 25.18)	2.48 (0.18 to 31.74)	0.75 (0.06 to 9.15)
0.51 (0.13 to 2.1)	0.45 (0.10 to 1.89)	0.64 (0.13 to 3.18)	0.76 (0.1 to 5.93)	0.34 (0.05 to 2.24)	0.51 (0.13 to 1.89)	0.56 (0.05 to 7.08)	Leukocyte immune therapy	0.59 (0.15 to 2.19)	0.37 (0.11 to 1.22)	0.86 (0.1 to 7.2)	1.39 (0.23 to 8.68)	0.42 (0.07 to 2.34)
0.87 (0.37 to 2.11)	0.76 (0.29 to 2.02)	1.08 (0.33 to 3.72)	1.30 (0.23 to 7.84)	0.59 (0.12 to 2.78)	0.86 (0.37 to 1.94)	0.95 (0.10 to 9.71)	1.70 (0.46 to 6.50)	LMWH	0.64 (0.35 to 1.14)	1.46 (0.23 to 9.22)	2.36 (0.59 to 9.81)	0.71 (0.18 to 2.81)
1.37 (0.67 to 2.91)	1.20 (0.53 to 2.75)	1.70 (0.61 to 5.04)	2.05 (0.39 to 10.93)	0.94 (0.21 to 3.95)	1.35 (0.75 to 2.42)	1.50 (0.17 to 14.19)	2.68 (0.82 to 8.93)	1.57 (0.88 to 2.82)	Placebo	2.3 (0.4 to 13.33)	3.7 (0.95 to 15.13)	1.12 (0.32 to 3.88)
0.60 (0.09 to 4.00)	0.52 (0.08 to 3.60)	0.74 (0.10 to 5.68)	0.89 (0.08 to 9.70)	0.41 (0.04 to 3.81)	0.59 (0.09 to 3.66)	0.65 (0.04 to 11.09)	1.17 (0.14 to 9.70)	0.68 (0.11 to 4.3)	0.44 (0.08 to 2.49)	Prednisolone	1.62 (0.17 to 14.7)	0.49 (0.06 to 4.12)
0.37 (0.08 to 1.75)	0.32 (0.07 to 1.61)	0.46 (0.08 to 2.69)	0.55 (0.06 to 4.65)	0.25 (0.03 to 1.82)	0.36 (0.08 to 1.61)	0.40 (0.03 to 5.67)	0.72 (0.12 to 4.43)	0.42 (0.1 to 1.69)	0.27 (0.07 to 1.06)	0.62 (0.07 to 5.96)	Prednisone plus progesterone plus aspirin	0.3 (0.05 to 1.95)
1.22 (0.29 to 5.34)	1.07 (0.24 to 4.92)	1.52 (0.31 to 8.03)	1.85 (0.23 to 14.74)	0.83 (0.12 to 5.57)	1.21 (0.30 to 4.75)	1.34 (0.11 to 17.41)	2.41 (0.43 to 13.41)	1.4 (0.36 to 5.57)	0.89 (0.26 to 3.14)	2.05 (0.24 to 17.99)	3.34 (0.51 to 21.55)	Progesterone
Proportion of participants in placebo group												
63 (95% CI 39 to 84)	58 (95% CI 33 to 82)	71 (95% CI 46 to 91)	86 (95% CI 73 to 95)	41 (95% CI 31 to 51)	57 (95% CI 49 to 66)	89 (95% CI 79 to 99)	68 (95% CI 55 to 79)	77 (95% CI 69 to 83)	59 (95% CI 51 to 67)	60 (95% CI 37 to 81)	85 (95% CI 74 to 93)	66 (95% CI 61 to 70)
I-squared	I-squared	I-squared	I-squared	I-squared	I-squared	I-squared	I-squared	I-squared	I-squared	I-squared	I-squared	I-squared
95%	95%	84%	-	-	51%	-	-	83%	92%	-	-	-

Comparisons should be read from left to right. The comparative effectiveness estimate is located at the intersection of the column-defining and row-defining treatment. The values refer to odds ratios and corresponding 95% credible intervals. The interventions are ordered alphabetically. Odds ratios greater than 1 favor the column-defining intervention. Significant results are in bold. The proportion of women in the placebo group with a live birth is also presented. CI, confidence interval; G-CSF, granulocyte colony-stimulating factor; hCG, human chorionic gonadotropin; IVIG, intravenous immunoglobulin G; LMWH, low-molecular-weight heparin.

**Table 4:** Surface under the cumulative ranking curve (SUCRA) scores for live birth rate, expressed as a percentage, with higher values indicating a higher probability of an intervention being associated with a better outcome.

Intervention	SUCRA (%)
Prednisone plus progesterone plus aspirin	83
Leukocyte immune therapy	74
Prednisolone	65
hCG	61
G-CSF	56
LMWH	53
IVIG plus LMWH plus aspirin	48
Aspirin	45
IVIG	44
Aspirin plus LMWH	36
Progesterone	35
Intralipid	28
Placebo	22

G-CSF, granulocyte colony-stimulating factor; hCG, human chorionic gonadotropin; IVIG, intravenous immunoglobulin G; LMWH, low-molecular-weight heparin.

Based on 23 trials, the proportion of participants in the placebo group with a successful live birth was 59% (95% CI 51–67; *I*^2^ = 92%) (30, 32–36, 38, 41, 42, 45–47, 54–60, 62, 63, 65, 67) (Table 3).

### Miscarriage rate

**Figure 6:**
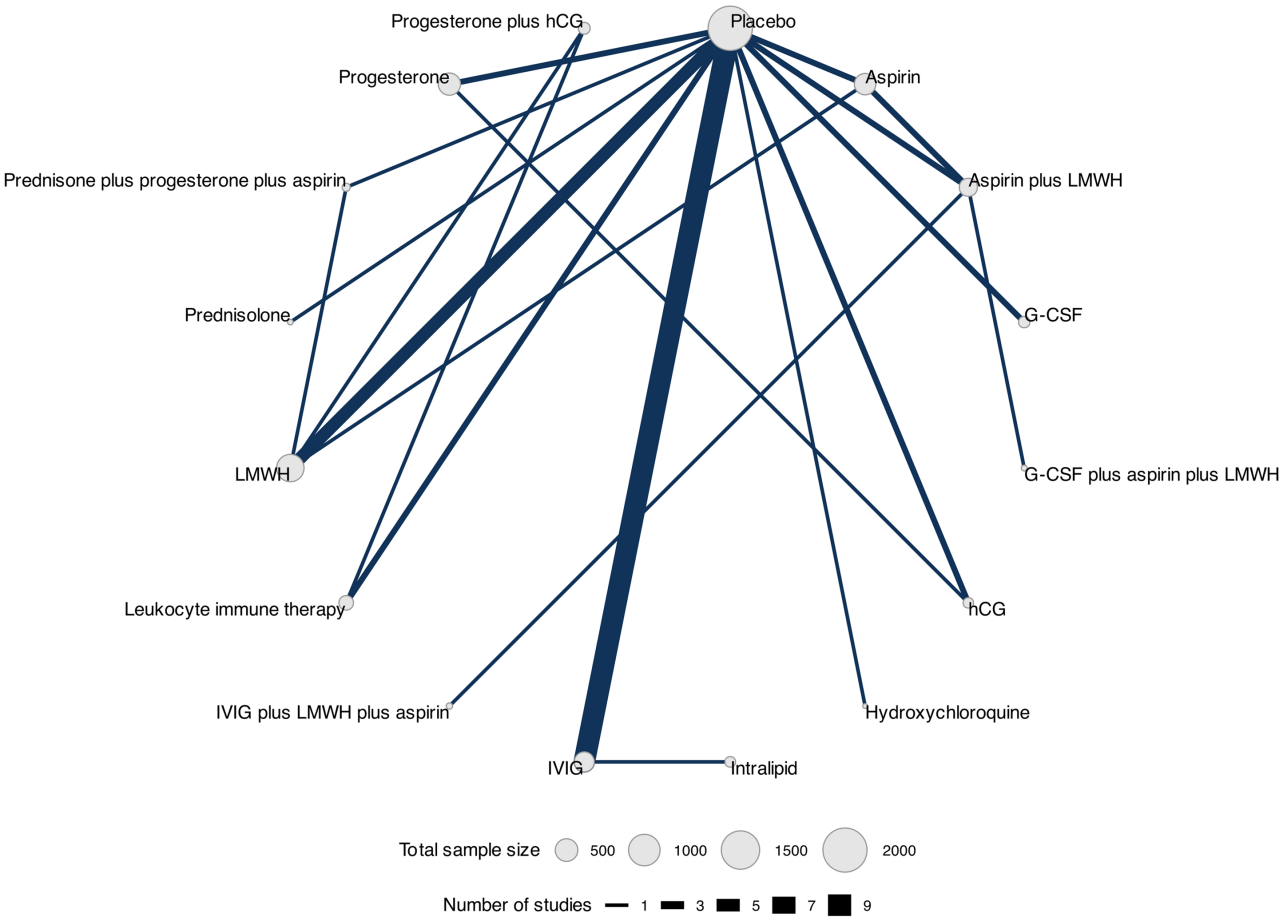
Network plot for miscarriage rate. Network plot showing comparisons in miscarriage rate between nodes (gray circles), each representing a therapeutic intervention. The size of each node is proportional to the total number of participants assigned to the intervention, and the width of each connecting line is proportional to the number of studies that have performed head-to-head comparisons between the two nodes.

The network plot showing the comparisons between therapeutic interventions for miscarriage rate is shown in Figure 6.

Data for this outcome were reported in 33 RCTs (30, 32–41, 45–49, 51–67) (5,125 participants) that compared 16 interventions. Three RCTs were three-arm trials (39, 41, 46) (Figure 3).

Overall, we found evidence of statistically significant differences between a single intervention versus placebo, namely, progesterone plus hCG, which presented increased odds of miscarriage (OR 3.83, 95% CrIs 1.04–14.38) as shown in Table 5 and Supplementary Figure S5. The three best-ranked interventions in terms of miscarriage rate (according to the SUCRA, where higher values indicate more certainty that the intervention is the best-ranked in the comparison) were prednisone plus progesterone plus aspirin (SUCRA = 81%), hydroxychloroquine (SUCRA = 79%), and intralipid (SUCRA = 65%) as shown in Table 6. We did not find evidence of publication bias for this outcome (Peters test, *p* = 0.065), and the certainty of evidence of the relative treatment effects varied from very low and low to moderate (Supplementary Table S4).

**Table 5:** League table showing the comparisons for the efficacy of each therapeutic intervention for miscarriage rate.

Aspirin	1.02 (0.43 to 2.43)	0.75(0.2 to 2.78)	0.91(0.14 to 5.99)	0.82(0.21 to 3.1)	0.28(0.01 to 3.75)	0.59(0.1 to 3.58)	0.96(0.38 to 2.54)	0.8(0.08 to 7.24)	1.17(0.34 to 4)	0.93(0.39 to 2.29)	1.37(0.65 to 3)	0.61(0.09 to 4.18)	0.37(0.07 to 1.8)	0.87(0.26 to 2.91)	5.3(1.21 to 23.84)
0.98(0.41 to 2.32)	Aspirin plus LMWH	0.74(0.18 to 2.9)	0.89(0.17 to 4.79)	0.81(0.2 to 3.24)	0.28(0.01 to 3.74)	0.58(0.09 to 3.77)	0.94(0.34 to 2.76)	0.79(0.1 to 6.02)	1.14(0.31 to 4.23)	0.91(0.34 to 2.54)	1.34(0.57 to 3.32)	0.59(0.09 to 4.22)	0.36(0.07 to 1.9)	0.86(0.23 to 3.08)	5.17(1.12 to 25.21)
1.33(0.36 to 4.95)	1.35(0.35 to 5.48)	G-CSF	1.21(0.14 to 10.7)	1.09(0.24 to 5.01)	0.37(0.02 to 5.41)	0.78(0.12 to 5.56)	1.27(0.39 to 4.38)	1.07(0.09 to 12.37)	1.55(0.38 to 6.55)	1.23(0.38 to 4.23)	1.82(0.64 to 5.4)	0.8(0.1 to 6.47)	0.49(0.08 to 2.92)	1.16(0.28 to 4.8)	7.01(1.31 to 38.97)
1.1(0.17 to 7.13)	1.12(0.21 to 5.95)	0.83(0.09 to 7.29)	G-CSF plus aspirin plus LMWH	0.91(0.1 to 8.07)	0.31(0.01 to 6.97)	0.65(0.05 to 7.8)	1.07(0.15 to 7.58)	0.88(0.06 to 12.06)	1.28(0.15 to 10.45)	1.03(0.14 to 7.23)	1.52(0.23 to 9.9)	0.66(0.05 to 8.75)	0.4(0.04 to 4.39)	0.96(0.11 to 7.78)	5.85(0.6 to 56.47)
1.21(0.32 to 4.71)	1.23(0.31 to 5.04)	0.91(0.2 to 4.19)	1.1(0.12 to 9.94)	hCG	0.34(0.01 to 4.98)	0.71(0.1 to 5.05)	1.16(0.35 to 4.09)	0.97(0.08 to 11.64)	1.42(0.33 to 6.02)	1.13(0.34 to 3.96)	1.66(0.57 to 5.06)	0.73(0.1 to 5.8)	0.45(0.07 to 2.72)	1.06(0.3 to 3.71)	6.4(1.17 to 35.89)
3.54(0.27 to 76.94)	3.63(0.27 to 80.74)	2.7(0.18 to 60.17)	3.27(0.14 to 106.93)	2.96(0.2 to 68.77)	Hydroxy chloroquine	2.09(0.12 to 59.09)	3.43(0.28 to 70.03)	2.89(0.1 to 114)	4.13(0.3 to 91.89)	3.32(0.27 to 67.08)	4.84(0.43 to 94.95)	2.18(0.11 to 68)	1.32(0.08 to 34.01)	3.09(0.23 to 66.54)	19.14(1.19 to 478.18)
1.69(0.28 to 10.08)	1.74(0.27 to 10.71)	1.28(0.18 to 8.62)	1.55(0.13 to 18.93)	1.4(0.2 to 9.66)	0.48(0.02 to 8.68)	Intralipid	1.62(0.37 to 7.56)	1.37(0.09 to 20.27)	1.98(0.3 to 12.63)	1.58(0.29 to 8.98)	2.33(0.47 to 12.02)	1.03(0.09 to 11.21)	0.62(0.07 to 5.5)	1.48(0.22 to 9.56)	8.98(1.13 to 74.2)
1.04(0.39 to 2.64)	1.06(0.36 to 2.94)	0.79(0.23 to 2.53)	0.94(0.13 to 6.84)	0.86(0.24 to 2.85)	0.29(0.01 to 3.56)	0.62(0.13 to 2.73)	IVIG	0.84(0.08 to 7.91)	1.22(0.39 to 3.6)	0.97(0.43 to 2.14)	1.42(0.81 to 2.49)	0.63(0.1 to 3.93)	0.38(0.08 to 1.72)	0.9(0.29 to 2.65)	5.5(1.3 to 22.86)
1.25(0.14 to 11.77)	1.26(0.17 to 10.09)	0.94(0.08 to 11.18)	1.13(0.08 to 16.21)	1.03(0.09 to 12.5)	0.35(0.01 to 10.19)	0.73(0.05 to 11.6)	1.2(0.13 to 12.25)	IVIG plus LMWH plus aspirin	1.45(0.13 to 16.98)	1.17(0.12 to 11.82)	1.72(0.19 to 16.33)	0.75(0.05 to 12.9)	0.46(0.03 to 6.53)	1.08(0.1 to 12.49)	6.58(0.51 to 88.76)
0.86(0.25 to 2.92)	0.88(0.24 to 3.21)	0.64(0.15 to 2.66)	0.78(0.1 to 6.51)	0.71(0.17 to 3.02)	0.24(0.01 to 3.34)	0.5(0.08 to 3.34)	0.82(0.28 to 2.59)	0.69(0.06 to 7.56)	Leukocyte immune therapy	0.8(0.28 to 2.33)	1.18(0.46 to 3.11)	0.52(0.07 to 3.85)	0.32(0.06 to 1.77)	0.75(0.19 to 2.88)	4.49(1.25 to 17.2)
1.07(0.44 to 2.58)	1.1(0.39 to 2.98)	0.81(0.24 to 2.64)	0.98(0.14 to 6.9)	0.88(0.25 to 2.98)	0.3(0.01 to 3.65)	0.63(0.11 to 3.49)	1.03(0.47 to 2.33)	0.86(0.08 to 8.14)	1.25(0.43 to 3.59)	LMWH	1.47(0.83 to 2.62)	0.65(0.1 to 4.07)	0.4(0.09 to 1.62)	0.93(0.3 to 2.79)	5.63(1.59 to 20.75)
0.73(0.33 to 1.54)	0.75(0.3 to 1.77)	0.55(0.19 to 1.55)	0.66(0.1 to 4.37)	0.6(0.2 to 1.75)	0.21(0.01 to 2.34)	0.43(0.08 to 2.13)	0.7(0.4 to 1.23)	0.58(0.06 to 5.22)	0.85(0.32 to 2.19)	0.68(0.38 to 1.2)	Placebo	0.44(0.08 to 2.53)	0.27(0.06 to 1.11)	0.64(0.24 to 1.6)	3.83(1.04 to 14.38)
1.65(0.24 to 10.97)	1.69(0.24 to 11.71)	1.24(0.15 to 9.58)	1.51(0.11 to 19.6)	1.36(0.17 to 10.38)	0.46(0.01 to 9.38)	0.97(0.09 to 10.63)	1.59(0.25 to 10.09)	1.33(0.08 to 22.13)	1.92(0.26 to 14.18)	1.54(0.25 to 9.72)	2.26(0.4 to 13.07)	Prednisolone	0.6(0.06 to 5.86)	1.44(0.19 to 10.58)	8.71(0.97 to 78.26)
2.7(0.56 to 13.78)	2.78(0.53 to 14.81)	2.05(0.34 to 12.16)	2.47(0.23 to 26.08)	2.24(0.37 to 13.52)	0.76(0.03 to 13.23)	1.61(0.18 to 13.88)	2.62(0.58 to 12.62)	2.19(0.15 to 30.41)	3.17(0.57 to 17.74)	2.53(0.62 to 10.97)	3.72(0.9 to 16.02)	1.65(0.17 to 16.16)	Prednisone plus progesterone plus aspirin	2.38(0.43 to 12.85)	14.33(2.16 to 97.74)
1.15(0.34 to 3.91)	1.17(0.32 to 4.38)	0.87(0.21 to 3.61)	1.04(0.13 to 8.94)	0.95(0.27 to 3.28)	0.32(0.02 to 4.44)	0.68(0.1 to 4.51)	1.11(0.38 to 3.4)	0.92(0.08 to 10.34)	1.34(0.35 to 5.22)	1.07(0.36 to 3.32)	1.57(0.63 to 4.21)	0.7(0.09 to 5.31)	0.42(0.08 to 2.34)	Progesterone	6.08(1.21 to 31.99)
**0.19(0.04 to 0.82)**	**0.19(0.04 to 0.9)**	**0.14(0.03 to 0.76)**	0.17(0.02 to 1.68)	**0.16(0.03 to 0.86)**	**0.05(0 to 0.84)**	**0.11(0.01 to 0.89)**	**0.18(0.04 to 0.77)**	0.15(0.01 to 1.97)	**0.22(0.06 to 0.8)**	**0.18(0.05 to 0.63)**	**0.26(0.07 to 0.96)**	0.11(0.01 to 1.03)	**0.07(0.01 to 0.46)**	**0.16(0.03 to 0.83)**	Progesterone plus hCG
Proportion of participants in placebo group															
23(95% CI 15 to 31)	29 (95% CI 14 to 45)	27(95% CI11 to 48)	35(95% CI 16 to 56)	16(95% CI 9 to 25)	8(95% CI 0 to 30)	9(95% CI 4 to 16)	29(95% CI 23 to 37)	11(95% CI 1 to 25)	29(95% CI 3 to 68)	19(95% CI 15 to 25)	35(95% CI 30 to 42)	4(95% CI 19 to 63)	13(95% CI 5 to 24)	23(95% CI 7 to 43)	28(95% CI 20 to 36)
I-squared	I-squared	I-squared	I-squared	I-squared	I-squared	I-squared	I-squared	I-squared	I-squared	I-squared	I-squared	I-squared	I-squared	I-squared	I-squared
75%	89%	78%	-	0%	-	-	62%	-	97%	69%	86%	-	-	93%	93%

Comparisons should be read from left to right. The comparative effectiveness estimate is located at the intersection of the column-defining treatment and the row-defining treatment. The values refer to odds ratios and corresponding 95% credible intervals. The interventions are ordered alphabetically. Odds ratios lower than 1 favor the column-defining intervention. Significant results are in bold. The proportion of women in the placebo group who had experienced a miscarriage is also presented. CI, confidence interval; G-CSF, granulocyte colony-stimulating factor; hCG, human chorionic gonadotropin; IVIG, intravenous immunoglobulin G; LMWH, low-molecular-weight heparin.

**Table 6:** Surface under the cumulative ranking curve (SUCRA) scores for miscarriage rate, expressed as a percentage, with higher values indicating a higher probability of an intervention being associated with a better outcome.

Intervention	SUCRA (%)
Prednisone plus progesterone plus aspirin	81
Hydroxychloroquine	79
Intralipid	65
Prednisolone	63
G-CSF	58
hCG	54
IVIG plus LMWH plus aspirin	53
Progesterone	52
G-CSF plus aspirin plus LMWH	49
LMWH	49
IVIG	47
Aspirin	44
Aspirin plus LMWH	43
Leukocyte immune therapy	37
Placebo	24
Progesterone plus hCG	2

G-CSF, granulocyte colony-stimulating factor; hCG, human chorionic gonadotropin; IVIG, intravenous immunoglobulin G; LMWH, low-molecular-weight heparin.

Based on 26 trials, the proportion of participants in the placebo group who underwent a miscarriage was 35% (95% CI 30–42; *I*^2^ = 86%) (30, 32–36, 38, 39, 41, 45–47, 51, 53–63, 65, 67) (Table 5).

### Trial discontinuation

**Figure 7:**
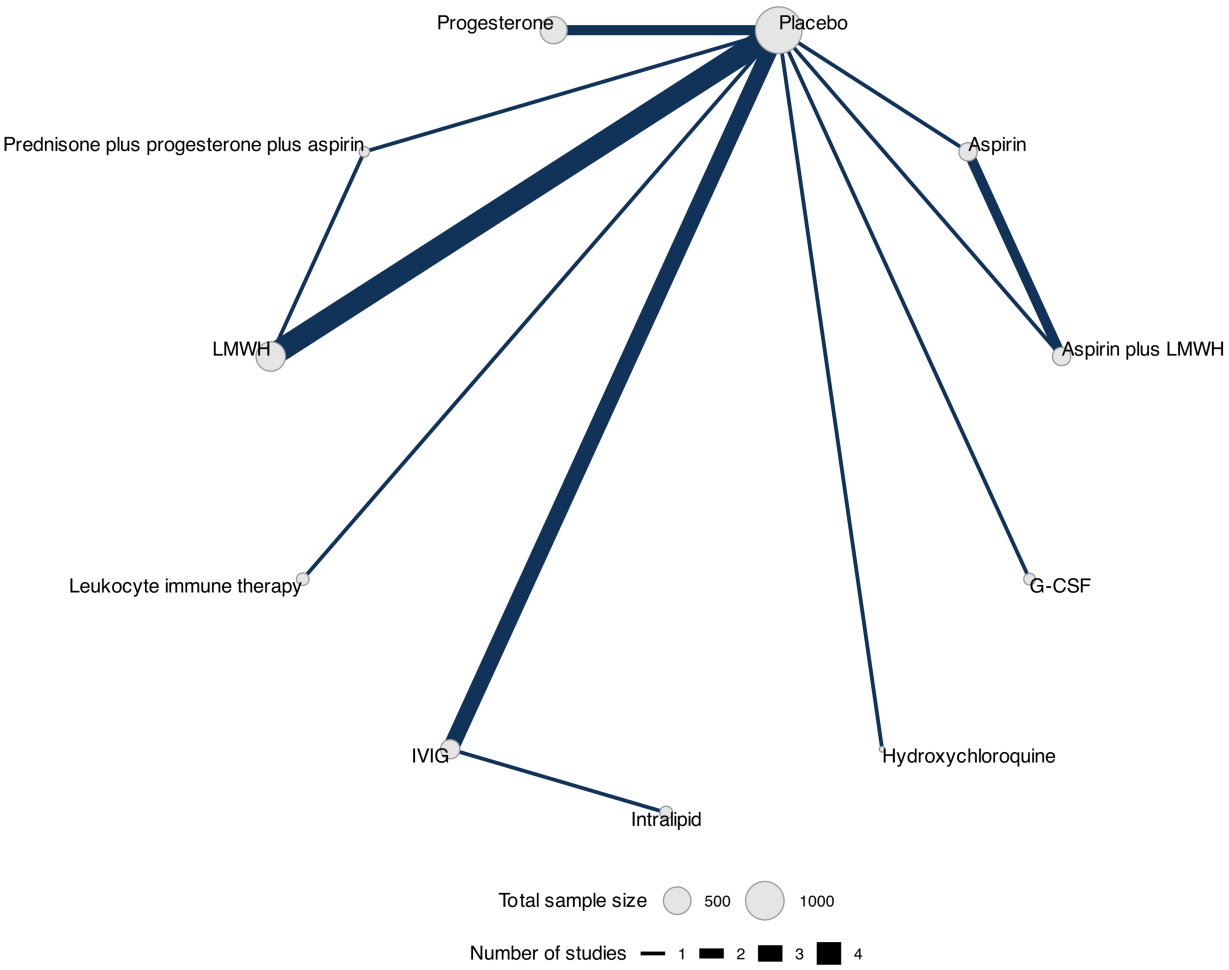
Network plot for trial discontinuation. Network plot showing comparisons in trial discontinuation between nodes (gray circles), each representing a therapeutic intervention. The size of each node is proportional to the total number of participants assigned to the intervention, and the width of each connecting line is proportional to the number of studies that have performed head-to-head comparisons between the two nodes.

The network plot showing the comparisons between therapeutic interventions for trial discontinuation is shown in Figure 7.

Data for this outcome were reported in 15 RCTs (32, 35, 38, 41, 44, 46, 49–51, 53, 54, 58–60, 65) (3,525 participants) that compared 11 interventions. Two RCTs were three-arm trials (41, 46) (Figure 4).

We found no evidence of statistically significant differences between interventions in patients who discontinued participating in the trial (Table 7 and Supplementary Figure S6). The three best-ranked interventions regarding trial discontinuation were LMWH (SUCRA = 74%), G-CSF (SUCRA = 72%), and leukocyte immune therapy (SUCRA = 68%) as shown in Table 8. We did not find any evidence of publication bias for this outcome (Peters test, *p* = 0.32), and the certainty of evidence of the relative treatment effects varied from low to moderate (Supplementary Table S5).

**Table 7:** League table showing the comparisons for the efficacy of each therapeutic intervention for trial discontinuation

Aspirin	1.05 (0.46 to 2.27)	0.66 (0.12 to 3.47)	1.26 (0.05 to 37.23)	1.51 (0.33 to 6.92)	1.29 (0.37 to 4.55)	0.74 (0.14 to 3.61)	0.72 (0.22 to 2.3)	1.03 (0.41 to 2.48)	4.05 (0.73 to 27.04)	1.15 (0.32 to 4.02)
0.95 (0.44 to 2.17)	Aspirin plus LMWH	0.62 (0.11 to 3.43)	1.21 (0.05 to 35.59)	1.43 (0.32 to 6.84)	1.22 (0.36 to 4.55)	0.71 (0.13 to 3.46)	0.68 (0.22 to 2.27)	0.98 (0.41 to 2.44)	3.86 (0.7 to 25.63)	1.09 (0.32 to 3.96)
1.53 (0.29 to 8.69)	1.6 (0.29 to 9.08)	G-CSF	1.94 (0.07 to 65.66)	2.3 (0.36 to 15.84)	1.97 (0.37 to 11.1)	1.13 (0.16 to 8.23)	1.09 (0.22 to 5.81)	1.58 (0.37 to 7.05)	6.19 (0.81 to 56.26)	1.76 (0.33 to 10.22)
0.79 (0.03 to 20.38)	0.83 (0.03 to 21.3)	0.51 (0.02 to 15)	Hydroxy chloroquine	1.18 (0.04 to 33.43)	1.02 (0.03 to 25.11)	0.59 (0.02 to 17.11)	0.57 (0.02 to 13.82)	0.81 (0.03 to 17.92)	3.16 (0.09 to 107.21)	0.9 (0.03 to 23.08)
0.66 (0.14 to 3.05)	0.7 (0.15 to 3.12)	0.44 (0.06 to 2.81)	0.85 (0.03 to 28.29)	Intralipid	0.86 (0.36 to 2.04)	0.49 (0.08 to 2.95)	0.48 (0.11 to 2.08)	0.69 (0.2 to 2.34)	2.7 (0.39 to 20.64)	0.76 (0.17 to 3.45)
0.78 (0.22 to 2.73)	0.82 (0.22 to 2.76)	0.51 (0.09 to 2.67)	0.98 (0.04 to 30.03)	1.16 (0.49 to 2.79)	IVIG	0.58 (0.11 to 2.74)	0.56 (0.17 to 1.8)	0.8 (0.32 to 1.92)	3.13 (0.55 to 20.36)	0.89 (0.25 to 3.07)
1.35 (0.28 to 7.16)	1.41 (0.29 to 7.44)	0.89 (0.12 to 6.27)	1.71 (0.06 to 60.67)	2.03 (0.34 to 12.78)	1.74 (0.37 to 9.18)	Leukocyte immune therapy	0.98 (0.21 to 4.73)	1.39 (0.38 to 5.49)	5.47 (0.79 to 46.05)	1.56 (0.32 to 7.97)
1.38 (0.44 to 4.47)	1.46 (0.44 to 4.49)	0.91 (0.17 to 4.46)	1.75 (0.07 to 49.34)	2.08 (0.48 to 9.02)	1.78 (0.56 to 5.87)	1.02 (0.21 to 4.66)	LMWH	1.43 (0.67 to 3.02)	5.55 (1.3 to 28.72)	1.59 (0.49 to 5.12)
0.97 (0.4 to 2.45)	1.02 (0.41 to 2.45)	0.63 (0.14 to 2.68)	1.24 (0.06 to 32.6)	1.46 (0.43 to 5.11)	1.25 (0.52 to 3.11)	0.72 (0.18 to 2.66)	0.7 (0.33 to 1.49)	Placebo	3.9 (0.92 to 21.09)	1.12 (0.46 to 2.76)
0.25 (0.04 to 1.37)	0.26 (0.04 to 1.43)	0.16 (0.02 to 1.24)	0.32 (0.01 to 11.15)	0.37 (0.05 to 2.53)	0.32 (0.05 to 1.82)	0.18 (0.02 to 1.26)	0.18 (0.03 to 0.77)	0.26 (0.05 to 1.08)	Prednisone plus progesterone plus aspirin	0.28 (0.04 to 1.54)
0.87 (0.25 to 3.11)	0.91 (0.25 to 3.12)	0.57 (0.1 to 3.07)	1.11 (0.04 to 33.65)	1.31 (0.29 to 6.04)	1.12 (0.33 to 3.99)	0.64 (0.13 to 3.16)	0.63 (0.2 to 2.04)	0.9 (0.36 to 2.18)	3.51 (0.65 to 23.22)	Progesterone
Proportion of participants in placebo group										
9 (95% CI 3 to 18)	9 (95% CI 6 to 25)	5 (95% CI 1 to 12)	7 (95% CI 0 to 28)	73 (95% CI 64 to 81)	26 (95% CI 3 to 61)	5 (95% CI 2 to 11)	2 (95% CI 0 to 6)	6 (95% CI 2 to 11)	12 (95% CI 5 to 21)	4 (95% CI 0 to 11)
I-squared	I-squared	I-squared	I-squared	I-squared	I-squared	I-squared	I-squared	I-squared	I-squared	I-squared
70%	89%	-	-	-	97%	-	75%	84%	-	82%

Comparisons should be read from left to right. The comparative effectiveness estimate is located at the intersection of the column-defining and row-defining treatment. The values refer to odds ratios and corresponding 95% credible intervals. The interventions are ordered alphabetically. Odds ratios greater than 1 favor the column-defining intervention. Significant results are in bold. The proportion of women in the placebo group who had discontinued from a trial is also presented. CI, confidence interval; G-CSF, granulocyte colony-stimulating factor; IVIG, intravenous immunoglobulin G; LMWH, low-molecular-weight heparin.

**Table 8:** Surface under the cumulative ranking curve (SUCRA) scores for trial discontinuation, expressed as a percentage, with higher values indicating a higher probability of an intervention being associated with a better outcome.

Intervention	SUCRA (%)
LMWH	74
G-CSF	72
Leukocyte immune therapy	68
Aspirin	55
Placebo	53
Aspirin plus LMWH	52
Hydroxychloroquine	47
Progesterone	47
IVIG	41
Intralipid	34
Prednisone plus progesterone plus aspirin	8

G-CSF, granulocyte colony-stimulating factor; IVIG, intravenous immunoglobulin G; LMWH, low-molecular-weight heparin.

Based on 13 trials, 6% of the participants in the placebo group discontinued the trials (95% CI 2–11, *I*^2^ = 84%) (32, 35, 38, 41, 44, 46, 51, 53, 54, 58–60, 65) (Table 7).

## Discussion

We applied NMA to compare the efficacy of several prophylactic therapeutic interventions in women with idiopathic RPL by synthesizing data from published RCTs. The primary outcomes included live birth and miscarriage rates, and the secondary outcomes were serious adverse/adverse events and trial discontinuation.

No significant differences between any of the interventions, including the placebo, were found concerning live birth rates. Nevertheless, based on this outcome, prednisone plus progesterone plus aspirin (first), leukocyte immune therapy (second), and prednisolone (third) were ranked among the top three interventions in terms of best live birth rates.

Regarding miscarriage rates, only progesterone plus hCG showed significant differences compared to other interventions, including placebos, as this treatment combination produced an increase in the odds of miscarriage. The three best-ranked interventions regarding this outcome were prednisone plus progesterone plus aspirin (first), hydroxychloroquine (second), and intralipid (third).

Regarding secondary outcomes, due to a lack of data concerning serious adverse/adverse events, we could not apply NMA to these outcomes.

Regarding trial discontinuations, we did not find statistically significant differences between the interventions assessed. This finding may reflect an equivalent tolerability to all assessed interventions in this specific population, which includes women who are highly motivated to adhere to a therapeutic option. However, the results do not reflect the totality of the available data. Following the standard recommendations for NMA, we excluded data from trials with no events in any trial arm.

The present analysis is comprehensive and introduces the idea of reevaluating the effects of different management therapeutic intervention options on both live birth and miscarriage rates and adverse events associated with the therapeutic interventions in women with idiopathic RPL. Furthermore, the certainty of evidence of the relative treatment effects varied from low to moderate for the live birth rate and trial discontinuation outcomes and from very low to moderate for the miscarriage rate outcome. These results emphasize the need for additional studies addressing the efficacy of therapeutic interventions used in idiopathic RPL in clinical trial settings. Our analysis found no significant improvements in live birth rates with any intervention, a finding that underscores the need for further research into effective therapies for idiopathic RPL.

This review was the first NMA that included data from published RCTs. Our review presents a thorough comparison of the efficacy of several therapeutic interventions in women with idiopathic RPL.

An earlier systematic review and meta-analyses examined the effects of different therapeutic interventions on live birth rates and adverse events associated with the interventions in women with idiopathic RPL (19). In these meta-analyses, the authors searched for RCTs until 2017 and included 21 studies (3,984 patients) assessing the effect of acetylsalicylic acid, LMWH, progesterone, IVIG, and leukocyte immune therapy in women who underwent three or more idiopathic RPL (19). The results from these meta-analyses indicated that no significant differences were found in live birth rates between the different therapeutic interventions, except for leukocyte immune therapy (risk ratio [RR] 1.8, 95% CI 1.34–2.41) and the use of progesterone initiated in the luteal phase (RR 1.18, 95% CI 1.09–1.27), which may be effective in improving live birth rates.(19) In contrast to our review and NMA, which included 38 eligible studies (6,379 participants), the authors did not include any RCT in which the intervention included corticosteroids, hydroxychloroquine, intralipid, G-CSF, and/or hCG. Furthermore, no serious adverse events or side effects were reported for the interventions analyzed in this meta-analysis (19). In contrast, in our study, conducting an NMA was not possible due to the scarcity of data regarding both serious adverse/adverse events in the included RCTs.

Using this network approach, which allows direct and indirect comparisons and subsequent ranking of the therapeutic interventions, we did not find any effective intervention that was capable of improving the live birth rate or reducing the miscarriage rate in women with idiopathic RPL. In contrast, we found evidence of statistically significant differences between the placebo and a single intervention, namely, progesterone combined with hCG, which was associated with an increase in the odds of miscarriage (OR 3.83, 95% CrIs 1.04–14.38).

Unfortunately, despite numerous advances in this field, several pregnancies still end in miscarriage; thus, no satisfactory explanation can be provided to approximately 50% of women with idiopathic RPL (1, 2, 11, 17). Therefore, a high number of women with idiopathic RPL are often exposed to therapeutic interventions based on theoretical hypotheses without proven efficacy (22). On the other hand, the prognosis is often favorable, and approximately two-thirds of women with a history of RPL may be able to undergo a subsequent pregnancy that results in a live birth even without therapeutic intervention and after being referred to a specialist (68).

Many empirical therapies aimed at reducing pro-inflammatory states and natural killer cell activity have been based on recent theories suggesting that immunological incompatibility at the maternal–fetal interface contributes to the pathophysiology of RPL (69, 70). The results of our NMA agree with other systematic reviews and meta-analyses that demonstrate that most of these immune therapies, which include corticosteroids, aspirin, LMWH, progesterone, hydroxychloroquine, IVIG, leukocyte immune therapy, intralipids, G-CSF, and tumor necrosis factor-alpha (TNF-*α*) antagonists, provide no significant and consistent beneficial effects over placebos in improving the live birth rates in women with RPL (19, 71–77).

Study strengths include a comprehensive search strategy and robust statistical analyses. We chose this recently described methodology in which data from randomized comparisons were combined to provide an internally consistent set of estimates while respecting the randomization of the evidence. This method may be a particularly useful tool in clinical decision-making scenarios. We believe that our NMA presents several additional strengths. To date, no systematic review and NMA of RCTs comparing the efficacy of therapeutic interventions for women with idiopathic RPL has been designed. We followed the PRISMA-NMA guidelines, and all results were reported according to the respective checklist (23). We conducted an extensive literature search with several updates to include all eligible trials containing high-quality data. Two independent reviewers extracted data and assessed the risk of bias using the Cochrane risk of bias tool. Discrepancies were resolved through discussion with a third reviewer. To attain the highest quality of evidence, this review included only RCTs, and the certainty of the generated evidence was assessed using the GRADE approach (29). We consider the inclusion of our team, which consisted of obstetricians, clinical pharmacologists, and librarians, as crucial for enhancing the integrity of our results and further validating the robustness of our network meta-analysis.

We acknowledge some limitations in our NMA. Two of the meta-analysis models assessed, namely, live birth rate and miscarriage rate, exhibited moderate-to-high levels of statistical heterogeneity, which may limit the generalizability of the findings. This heterogeneity likely stems from multiple factors, including variations in study design, different trial methodologies, and the complex interplay of genetic, immunological, and environmental influences on RPL. The presence of statistical heterogeneity underscores the need for caution when interpreting our results as the effectiveness of interventions may vary considerably depending on patient characteristics and study context. While subgroup analyses and meta-regression techniques can help explore potential sources of heterogeneity, they do not fully resolve the underlying uncertainty. Moreover, the sparse numbers of studies for each intervention in our systematic review preclude us from a rigorous assessment of this phenomenon. Future research should focus on identifying and characterizing specific subgroups of RPL patients to improve the precision and homogeneity of subsequent studies. In addition, the use of standardized definitions of RPL, rigorous study designs, and detailed reporting of patient characteristics will be essential for minimizing heterogeneity in future research and improving the reliability of findings in this complex field.

Most included studies did not provide data regarding serious adverse and/or adverse events; therefore, we could not assess the adverse events of the analyzed therapeutic interventions. Nevertheless, we presented the available data regarding these outcomes. In addition, the inclusion of studies with small samples could have raised the risk of bias in our NMA. Although we defined RPL as two or more clinically diagnosed miscarriages before 24 gestational weeks in this NMA, the heterogeneity of definitions and criteria applied by international guidelines for RPL in the different included RCTs can be considered a study limitation. The time at which the therapeutic interventions were initiated and their duration also differed in the included RCTs. Finally, our results were established based on both direct and indirect comparisons. Prospective RCTs should focus on direct comparisons of different therapeutic interventions, and future RCTs may further confirm the results of this NMA.

Furthermore, as already mentioned, the certainty of evidence of the relative treatment effects varied from low to moderate for the live birth rate and trial discontinuation outcomes and from very low to moderate for the miscarriage rate outcome, thus strengthening the urgent need for additional studies on the efficacy of therapeutic interventions used for idiopathic RPL in clinical trial settings.

## Conclusion

RPL is a traumatic life event that affects women’s health. An increasing number of work-up and therapeutic options are being offered to women with this highly heterogeneous condition. Our NMA suggests that none of the analyzed therapeutic interventions, including placebo, led to improvements in live birth rates or reductions in miscarriage rates in women with idiopathic RPL. This study highlights the lack of effective interventions for improving live birth rates in women with idiopathic RPL and emphasizes the need for continued research in this area. Additional studies on the efficacy of therapeutic interventions used in idiopathic RPL in clinical trial settings are urgently needed and must include investigating potential adverse events associated with these interventions. Future studies should focus on large-scale RCTs by directly comparing these interventions and assessing long-term outcomes.

## Data Availability

The original contributions presented in the study are included in the article/Supplementary material, further inquiries can be directed to the corresponding author/s.
