# Supplementary figures and images for: Efficacy of therapeutic interventions for idiopathic recurrent pregnancy loss: a systematic review and network meta-analysis
Source: Front Med (Lausanne). 2025 May 14;12:1569819. doi: 10.3389/fmed.2025.1569819 (PMC12116322; doi:10.3389/fmed.2025.1569819)

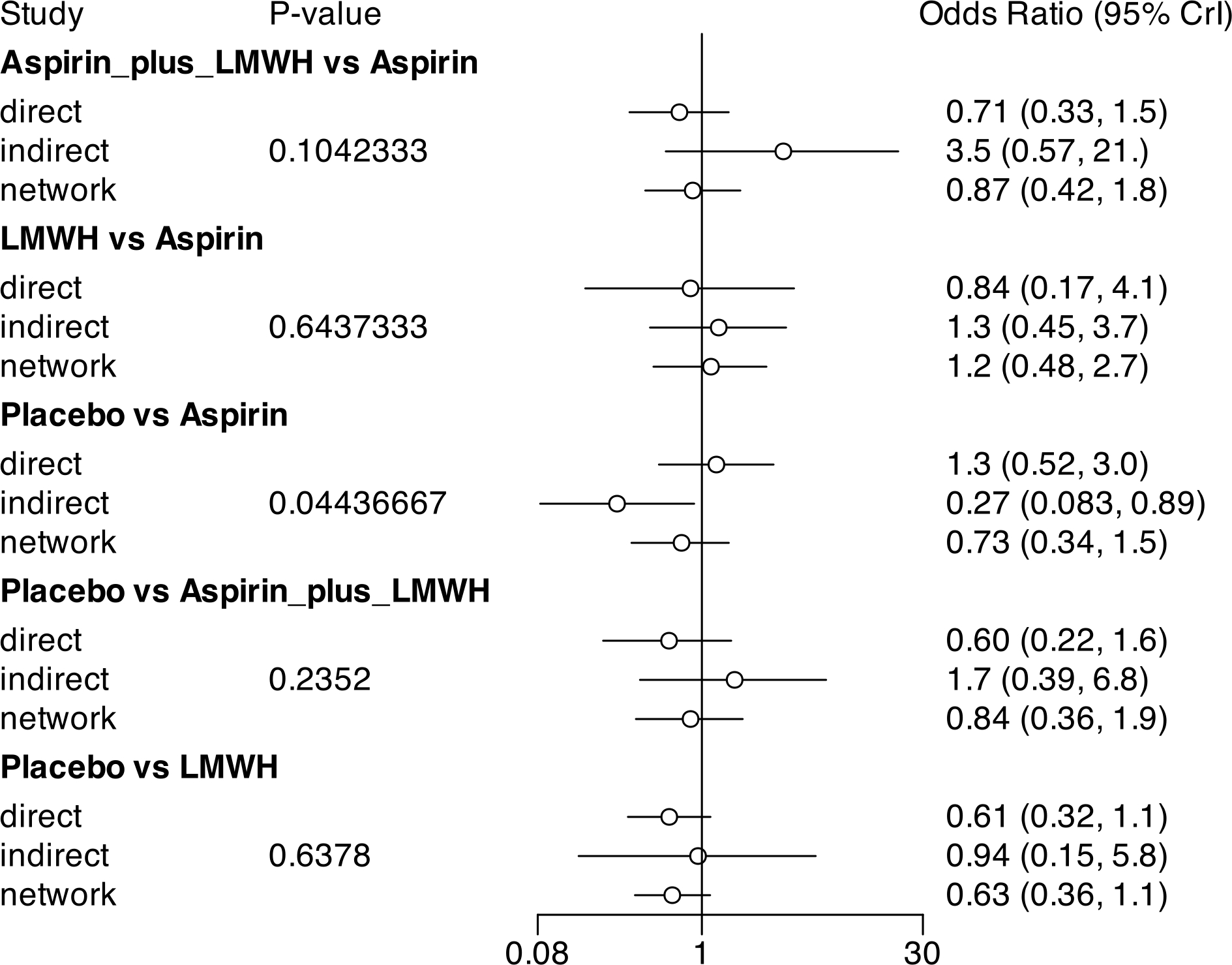

Supplement: Supplementary file 1 [file Image_1.JPEG]

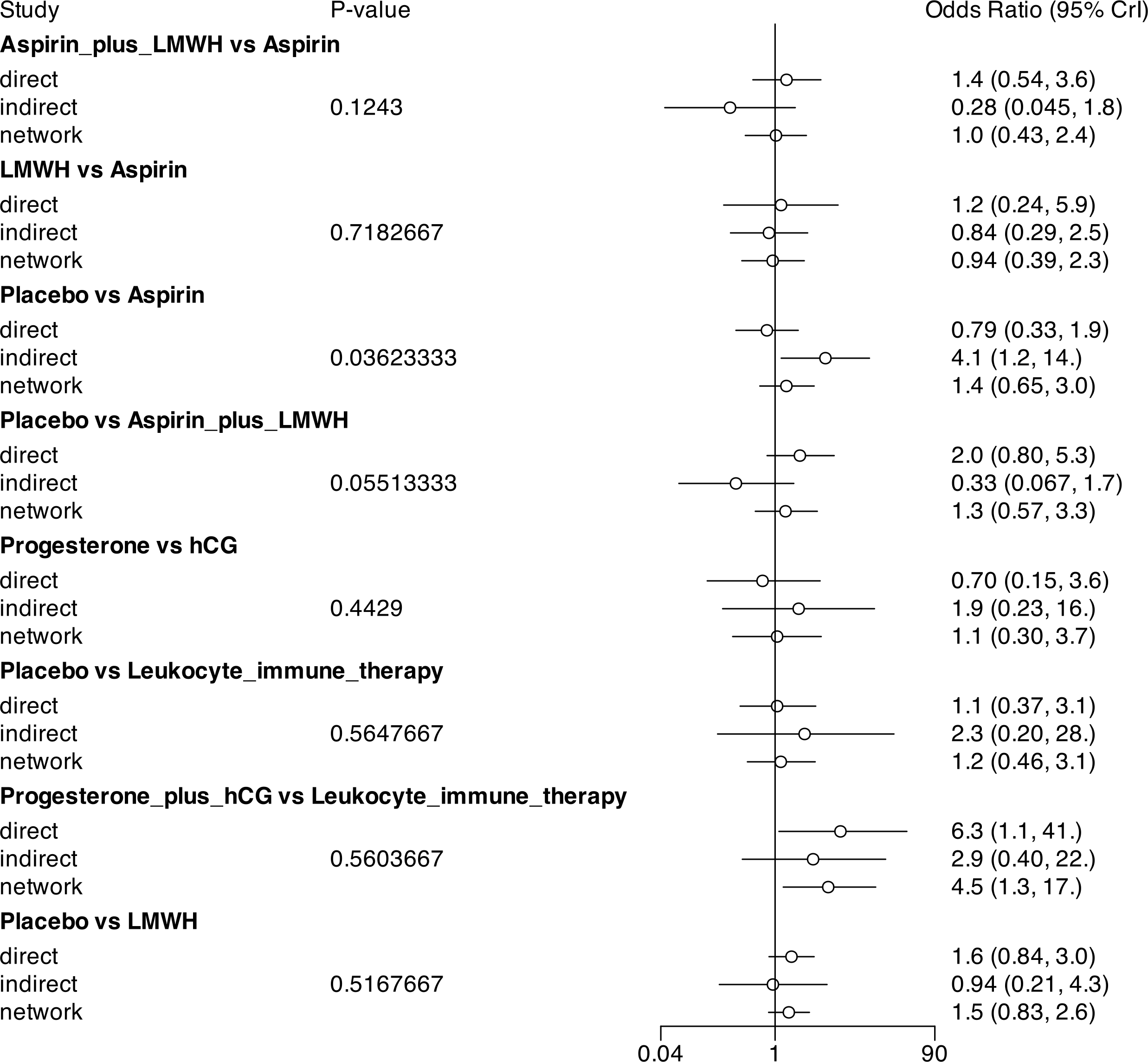

Supplement: Supplementary file 2 [file Image_2.JPEG]

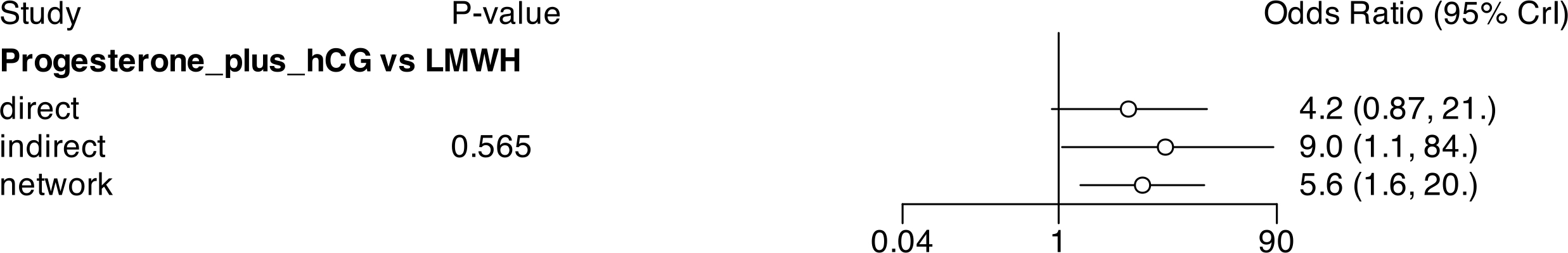

Supplement: Supplementary file 3 [file Image_3.JPEG]

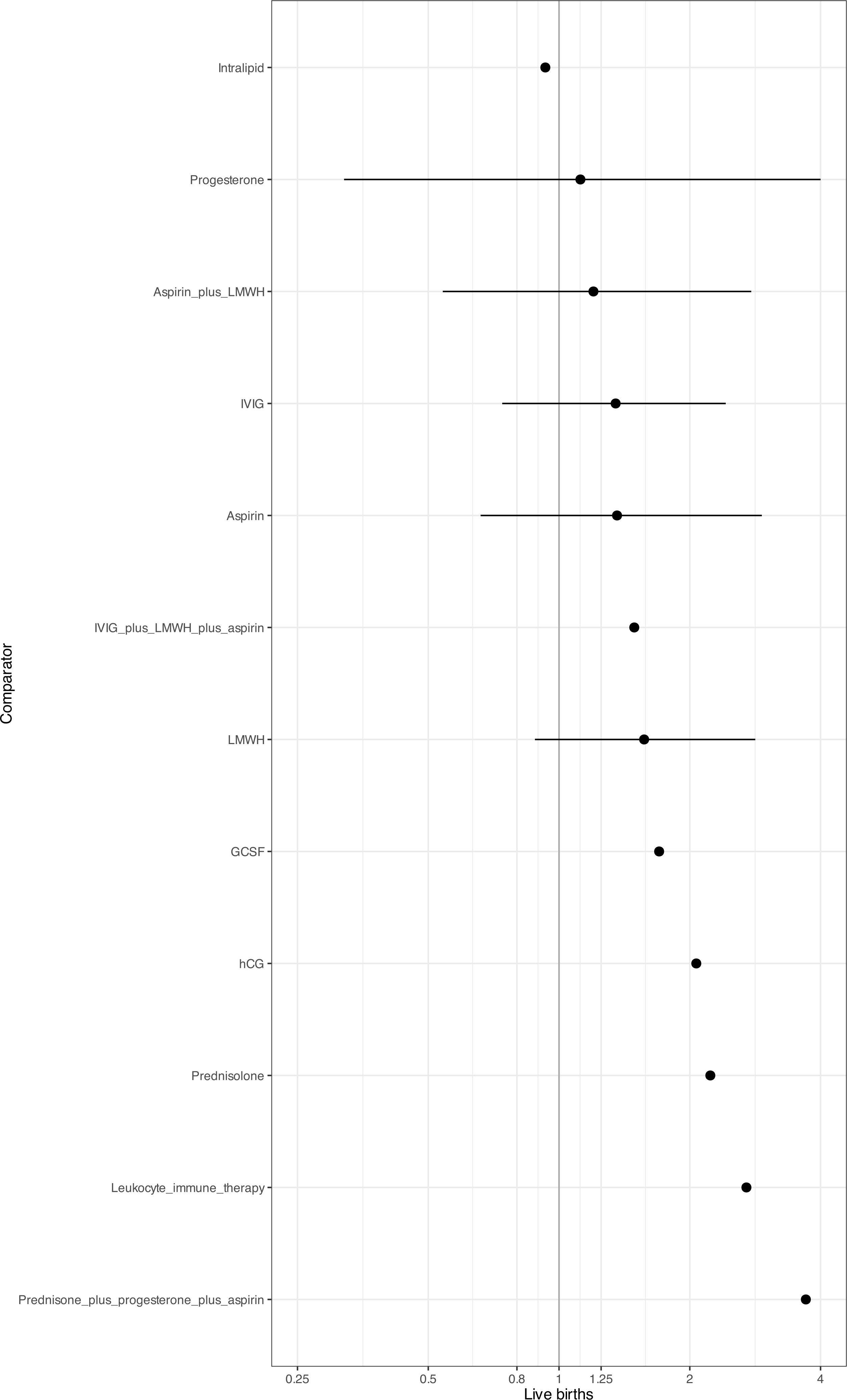

Supplement: Supplementary file 4 [file Image_4.JPEG]

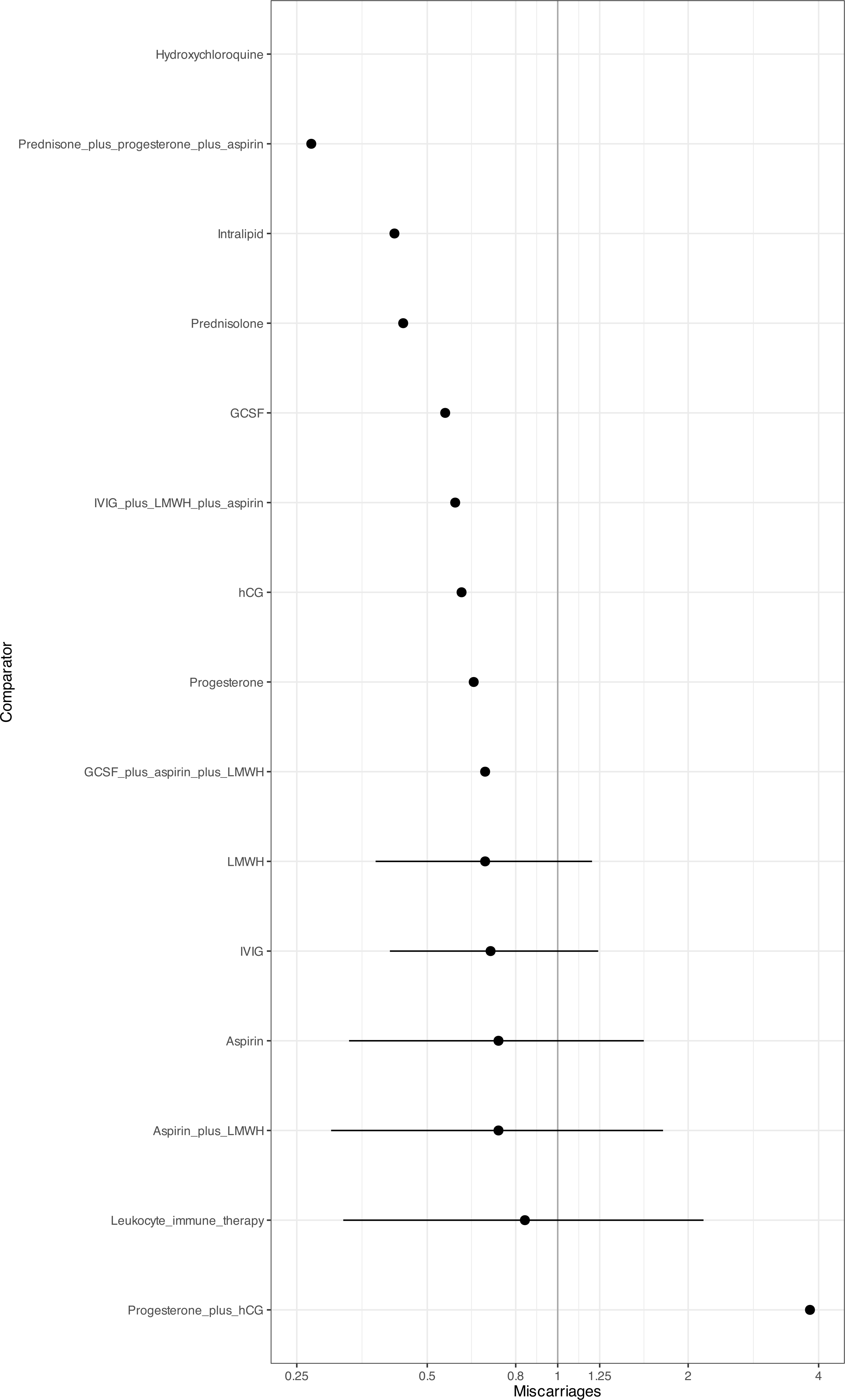

Supplement: Supplementary file 5 [file Image_5.JPEG]

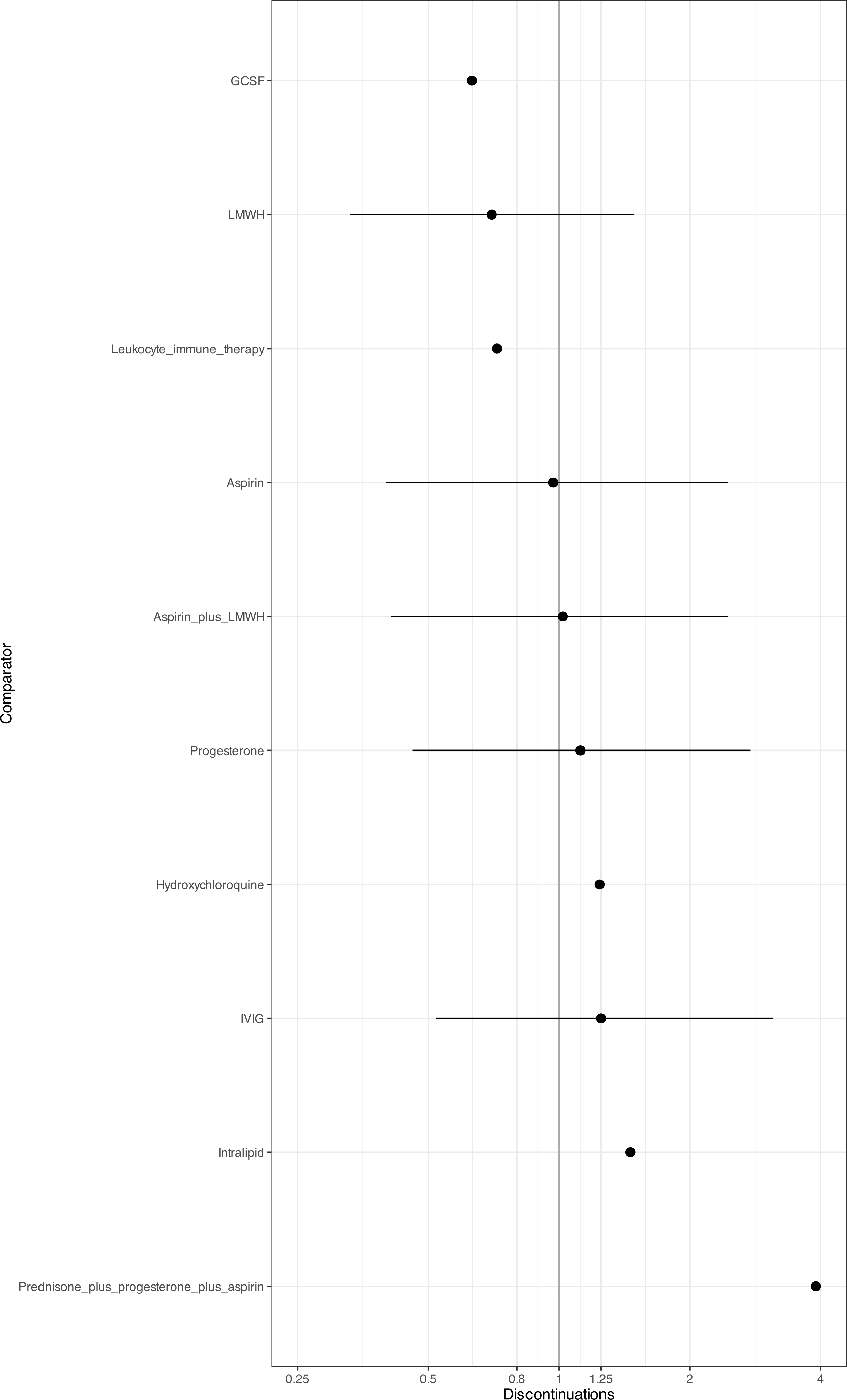

Supplement: Supplementary file 6 [file Image_6.JPEG]
