# Supplementary material for: Efficacy of therapeutic interventions for idiopathic recurrent pregnancy loss: a systematic review and network meta-analysis
Source: Front Med (Lausanne). 2025 May 14;12:1569819. doi: 10.3389/fmed.2025.1569819 (PMC12116322; doi:10.3389/fmed.2025.1569819)
Supplement: Supplementary file 8 [file Table_2.DOCX]

**Supplementary material**

**Supplementary Table S2.** Search strategy in PubMed, EMBASE, Cochrane Library, Scopus, and Web of Science.

| **Query** | **Search** |
| --- | --- |
| **PubMed** | *ALL FIELDS* |
| #1 | "abortion, habitual" OR "habitual abortion*" OR "recurrent miscarriage*" OR "recurrent abortion*" OR "recurrent pregnancy loss*" OR "spontaneous abortion*" OR "recurrent spontaneous abortion*" OR "repeated abortion*" OR "repeated miscarriage*" OR "repetitive miscarriage*" OR "repetitive abortion*"OR "successive abortion*" OR "successive pregnancy loss*" OR "recurrent reproductive failure" OR "pregnancy loss*" |
| #2 | idiopathic OR unexplained |
| #3 | "drug therap*" OR treatment* OR "acetylsalicylic acid" OR aspirin OR "ASA" OR anticoagulant OR heparin OR "low-molecular-weight heparin" OR "LMWH" OR "unfractionated heparin" OR "UFH" OR progesterone OR corticosteroids OR glucocorticoids OR "intravenous immunoglobulin" OR IvIg OR "leukocyte immune therapy" OR "leukocyte immunotherapy" OR "lymphocyte immunization therapy" OR "LIT" OR "paternal lymphocyte immunization" OR "human chorionic gonadotrophin" OR "hCG" OR "human chorionic gonadotropin" OR hydroxychloroquine OR "HCQ" OR "granulocyte colony-stimulating factor" OR "G-CSF" OR "intralipid therapy" OR "lipid emulsion" OR levothyroxine OR "folic acid" OR multivitamin OR "clomiphene citrate" OR sitagliptin OR metformin OR "vitamin D supplement*" |
| #4 | #1 AND #2 AND #3 |
| #5 | #1 AND #2 AND #3 (filters: Clinical Study, Clinical Trial, Controlled Clinical Trial, Randomized Controlled Trial, Humans) |
| **EMBASE** | *Title/Abstract/Author Supplied Keywords* |
| #1 | ((('habitual abortion':ab,ti,kw OR 'recurrent abortion':ab,ti,kw OR 'recurrent miscarriage':ab,ti,kw OR ‘recurrent pregnancy loss':ab,ti,kw OR 'spontaneous abortion':ab,ti,kw OR 'spontaneous recurrent abortion':ab,ti,kw OR 'repeated abortion':ab,ti,kw OR 'repeated miscarriage':ab,ti,kw OR 'repetitive miscarriage':ab,ti,kw OR 'repetitive abortion':ab,ti,kw OR 'sucessive abortion':ab,ti,kw OR 'successive pregnancy loss':ab,ti,kw OR 'recurrent reproductive failure':ab,ti,kw OR 'pregnancy loss':ab,ti,kw) AND (idiopathic:ab,ti,kw OR unexplained:ab,ti,kw) AND ('drug therapy':ab,ti,kw OR ‘treatment':ab,ti,kw OR 'acetylsalicylic acid':ab,ti,kw OR ‘aspirin':ab,ti,kw OR 'asa':ab,ti,kw OR 'anticoagulant':ab,ti,kw OR ‘heparin':ab,ti,kw OR 'low molecular weight heparin':ab,ti,kw OR ‘lmwh':ab,ti,kw OR 'unfractionated heparin':ab,ti,kw OR 'ufh':ab,ti,kw OR 'progesterone':ab,ti,kw OR 'corticosteroid':ab,ti,kw OR ‘glucocorticoid':ab,ti,kw OR 'intravenous immunoglobulin':ab,ti,kw OR ‘ivig':ab,ti,kw OR 'leukocyte immune therapy':ab,ti,kw OR 'leukocyte immunotherapy':ab,ti,kw OR 'leukocyte immunization therapy':ab,ti,kw OR ‘lit':ab,ti,kw OR 'paternal lymphocyte immunization':ab,ti,kw OR 'chorionic gonadotropin':ab,ti,kw OR 'hcg':ab,ti,kw OR 'granulocyte colony stimulating factor':ab,ti,kw OR 'g-csf':ab,ti,kw OR 'intralipid therapy':ab,ti,kw OR 'lipid emulsion':ab,ti,kw OR ‘levothyroxine':ab,ti,kw OR 'folic acid':ab,ti,kw OR ‘multivitamin':ab,ti,kw OR 'clomifene citrate':ab,ti,kw OR ‘sitagliptin':ab,ti,kw OR 'metformin':ab,ti,kw OR 'vitamin d supplementation':ab,ti,kw)) AND ('clinical article'/de OR 'clinical study'/de OR 'clinical trial'/de OR 'controlled clinical trial'/de OR ‘randomized controlled trial'/de)) AND 'human'/de |
| **Cochrane Library** | *Title, abstract, keyword* |
| #1 | ('habitual abortion' OR 'recurrent abortion' OR 'recurrent miscarriage' OR 'recurrent pregnancy loss' OR 'spontaneous abortion' OR 'spontaneous recurrent abortion' OR 'repeated abortion' OR 'repeated miscarriage' OR 'repetitive miscarriage' OR 'repetitive abortion' OR 'sucessive abortion' OR 'successive pregnancy loss' OR 'recurrent reproductive failure' OR 'pregnancy loss') in Title Abstract Keyword  AND (idiopathic OR unexplained) in Title Abstract Keyword AND ('drug therapy' OR 'treatment' OR 'acetylsalicylic acid' OR 'aspirin' OR 'asa' OR 'anticoagulant' OR 'heparin' OR 'low molecular weight heparin' OR 'lmwh' OR 'unfractionated heparin' OR 'ufh' OR 'progesterone' OR 'corticosteroid' OR 'glucocorticoid' OR 'intravenous immunoglobulin' OR 'ivig' OR 'leukocyte immune therapy' OR 'leukocyte immunotherapy' OR 'leukocyte immunization therapy' OR 'lit' OR 'paternal lymphocyte immunization' OR 'chorionic gonadotropin' OR 'hcg' OR 'granulocyte colony stimulating factor' OR 'g-csf' OR 'intralipid therapy' OR 'lipid emulsion' OR 'levothyroxine' OR 'folic acid' OR 'multivitamin' OR 'clomifene citrate' OR 'sitagliptin' OR 'metformin' OR 'vitamin d supplementation') AND ('clinical article' OR 'clinical study' OR 'clinical trial' OR 'controlled clinical trial' OR 'randomized controlled trial') in Title Abstract Keyword - (Word variations have been searched) |
| **Scopus** | *Article title, abstract, keywords* |
| #1 | "abortion, habitual" OR "habitual abortion*" OR "recurrent miscarriage*" OR "recurrent abortion*" OR "recurrent pregnancy loss*" OR "spontaneous abortion*" OR "recurrent spontaneous abortion*" OR "repeated abortion*" OR "repeated miscarriage*" OR "repetitive miscarriage*" OR "repetitive abortion*"OR "successive abortion*" OR "successive pregnancy loss*" OR "recurrent reproductive failure" OR "pregnancy loss*" |
| #2 | idiopathic OR unexplained |
| #3 | "drug therap*" OR treatment* OR "acetylsalicylic acid" OR aspirin OR "ASA" OR anticoagulant OR heparin OR "low-molecular-weight heparin" OR "LMWH" OR "unfractionated heparin" OR "UFH" OR progesterone OR corticosteroids OR glucocorticoids OR "intravenous immunoglobulin" OR IvIg OR "leukocyte immune therapy" OR "leukocyte immunotherapy" OR "lymphocyte immunization therapy" OR "LIT" OR "paternal lymphocyte immunization" OR "human chorionic gonadotrophin" OR "hCG" OR "human chorionic gonadotropin" OR hydroxychloroquine OR "HCQ" OR "granulocyte colony-stimulating factor" OR "G-CSF" OR "intralipid therapy" OR "lipid emulsion" OR levothyroxine OR "folic acid" OR multivitamin OR "clomiphene citrate" OR sitagliptin OR metformin OR "vitamin D supplement*" |
| #4 | #1 AND #2 AND #3 |
| #5 | #1 AND #2 AND #3 (filters: article) |
| **Web of Science** | *TOPIC - title, abstract, author keywords, and Keywords Plus* |
| #1 | "abortion, habitual" OR "habitual abortion*" OR "recurrent miscarriage*" OR "recurrent abortion*" OR "recurrent pregnancy loss*" OR "spontaneous abortion*" OR "recurrent spontaneous abortion*" OR "repeated abortion*" OR "repeated miscarriage*" OR "repetitive miscarriage*" OR "repetitive abortion*"OR "successive abortion*" OR "successive pregnancy loss*" OR "recurrent reproductive failure" OR "pregnancy loss*" |
| #2 | idiopathic OR unexplained |
| #3 | "drug therap*" OR treatment* OR "acetylsalicylic acid" OR aspirin OR "ASA" OR anticoagulant OR heparin OR "low-molecular-weight heparin" OR "LMWH" OR "unfractionated heparin" OR "UFH" OR progesterone OR corticosteroids OR glucocorticoids OR "intravenous immunoglobulin" OR IvIg OR "leukocyte immune therapy" OR "leukocyte immunotherapy" OR "lymphocyte immunization therapy" OR "LIT" OR "paternal lymphocyte immunization" OR "human chorionic gonadotrophin" OR "hCG" OR "human chorionic gonadotropin" OR hydroxychloroquine OR "HCQ" OR "granulocyte colony-stimulating factor" OR "G-CSF" OR "intralipid therapy" OR "lipid emulsion" OR levothyroxine OR "folic acid" OR multivitamin OR "clomiphene citrate" OR sitagliptin OR metformin OR "vitamin D supplement*" |
| #4 | #1 AND #2 AND #3 |
| #5 | #1 AND #2 AND #3 (filters: article) |
