# Supplementary material for: Efficacy of therapeutic interventions for idiopathic recurrent pregnancy loss: a systematic review and network meta-analysis
Source: Front Med (Lausanne). 2025 May 14;12:1569819. doi: 10.3389/fmed.2025.1569819 (PMC12116322; doi:10.3389/fmed.2025.1569819)
Supplement: Supplementary file 10 [file Table_4.DOCX]

**Supplementary material**

**Supplementary Table S4.** Certainty of evidence for the outcome miscarriage rate.

| **Outcome** | Miscarriage rate | | | 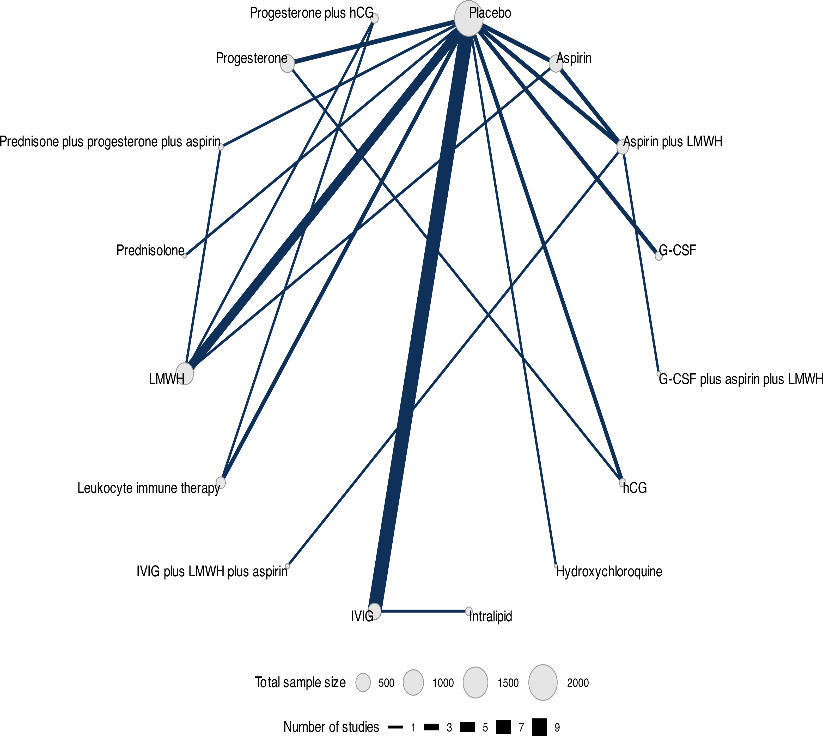 | | |
| --- | --- | --- | --- | --- | --- | --- |
| **Population** | Adult (i.e., ≥18 years) women with recurrent pregnancy loss | | |  |  |  |
| **Therapeutic interventions** | Aspirin  Aspirin plus LMWH  G-CSF  G-CSF plus aspirin plus LMWH  hCG  Hydroxychloroquine  Intralipid  IVIG  IVIG plus LMWH plus aspirin  Leukocyte immune therapy  LMWH  Prednisolone  Prednisone plus progesterone plus aspirin  Progesterone  Progesterone plus hCG | | |  |  |  |
| **Reference treatment** | Placebo | | |  |  |  |
| **Total studies:** 33  **Total participants:** 5125 | **Network estimate** | **Risk of bias** | **Indirectness** | **Inconsistency** | **Imprecision** | **Certainty** |
| **Aspirin versus placebo** | 0.73 (0.33 to 1.54) | Serious^1^ | Not serious | Serious^3^ | Serious^2^ | **VERY LOW**  Downgraded due to serious risk of bias, inconsistency, and imprecision |
| **Aspirin plus LMWH versus placebo** | 0.75 (0.30 to 1.77) | Serious^1^ | Not serious | Not serious | Serious^2^ | **LOW**  Downgraded due to serious risk of bias and imprecision |
| **G-CSF versus placebo** | 0.55 (0.19 to 1.55) | Not serious | Not serious | Not serious | Serious^2^ | **MODERATE**  Downgraded due to serious imprecision |
| **G-CSF plus aspirin plus LMWH versus placebo** | 0.66 (0.10 to 4.37) | Not serious | Not serious | Not serious |  | **MODERATE**  Downgraded due to serious imprecision |
| **hCG versus placebo** | 0.60 (0.20 to 1.75) | Not serious | Not serious | Not serious | Serious^2^ | **MODERATE**  Downgraded due to serious imprecision |
| **Hydroxychloroquine versus placebo** | 0.21 (0.01 to 2.34) | Not serious | Not serious | Not serious |  | **MODERATE**  Downgraded due to serious imprecision |
| **Intralipid versus placebo** | 0.43 (0.08 to 2.13) | Not serious | Not serious | Not serious | Serious^2^ | **MODERATE**  Downgraded due to serious imprecision |
| **IVIG versus placebo** | 0.70 (0.40 to 1.23) | Not serious | Not serious | Not serious | Serious^2^ | **MODERATE**  Downgraded due to serious imprecision |
| **IVIG plus LMWH plus aspirin versus placebo** | 0.58 (0.06 to 5.22) | Not serious | Not serious | Not serious | Serious^2^ | **MODERATE**  Downgraded due to serious imprecision |
| **Leukocyte immune therapy versus placebo** | 0.85 (0.32 to 2.19) | Not serious | Not serious | Not serious | Serious^2^ | **MODERATE**  Downgraded due to serious imprecision |
| **LMWH versus placebo** | 0.68 (0.38 to 1.20) | Serious^1^ | Not serious | Not serious | Serious^2^ | **LOW**  Downgraded due to serious risk of bias and imprecision |
| **Prednisolone versus placebo** | 0.44 (0.08 to 2.53) | Not serious | Not serious | Not serious | Serious^2^ | **MODERATE**  Downgraded due to serious imprecision |
| **Prednisone plus progesterone plus aspirin versus placebo** | 0.27 (0.06 to 1.11) | Not serious | Not serious | Not serious | Serious^2^ | **MODERATE**  Downgraded due to serious imprecision |
| **Progesterone versus placebo** | 0.64 (0.24 to 1.60) | Not serious | Not serious | Not serious | Serious^2^ | **MODERATE**  Downgraded due to serious imprecision |
| **Progesterone plus hCG versus placebo** | 3.83 (1.04 to 14.38) | Serious^1^ | Not serious | Not serious | Serious^2^ | **LOW**  Downgraded due to serious risk of bias and imprecision |
| **Table definitions**  *Solid lines represent direct comparison.  **Network estimates are reported as odds ratio with corresponding 95% credible intervals. Results are expressed as credible intervals as opposed to the confidence intervals since Bayesian analysis has been conducted, and these should be interpreted as the interval where there is a 95% probability that the values of the odds ratio will lie. | | | | | | |
| **GRADE Working Group Grades of Evidence (or certainty of the evidence)**  **High quality**: We are very confident the true effect lies close to that of the estimate of the effect.  **Moderate quality**: We are moderately confident in the effect estimate: The true effect is likely to be close to the estimate of effect, but there is a possibility that it is substantially different.  **Low quality**: Our confidence in the effect estimate is limited: The true effect may be substantially different from the estimate of effect.  **Very low quality**: We have very little confidence in the effect estimate: The true effect Is likely to be substantially different from the estimate of the effect. | | | | | | |
| **Explanatory footnotes**  **1:** Downgraded due to serious overall risk of bias across trials in the comparison in question.  **2:** Downgraded due to serious imprecision, namely with 95% credible intervals, which include widely disparate conclusions regarding the outcome in question.  **3:** Downgraded due to serious inconsistency, namely due to a suggestion of local inconsistency in node-split models, despite no evidence of inconsistency in global analysis. | | | | | | |

G-CSF, granulocyte colony-stimulating factor; hCG, human chorionic gonadotropin; IVIG, intravenous immunoglobulin G; LMWH, low-molecular-weight heparin.
