# Supplementary material for: Efficacy of therapeutic interventions for idiopathic recurrent pregnancy loss: a systematic review and network meta-analysis
Source: Front Med (Lausanne). 2025 May 14;12:1569819. doi: 10.3389/fmed.2025.1569819 (PMC12116322; doi:10.3389/fmed.2025.1569819)
Supplement: Supplementary file 11 [file Table_5.DOCX]

**Supplementary material**

**Supplementary Table S5.** Certainty of evidence for the outcome trial discontinuation.

| **Outcome** | Trial discontinuation | | | 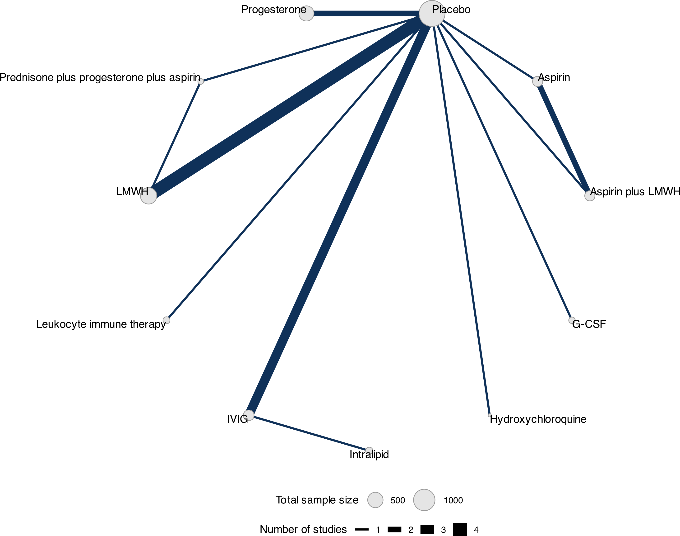 | | |
| --- | --- | --- | --- | --- | --- | --- |
| **Population** | Adult (i.e., ≥18 years) women with recurrent pregnancy loss | | |  |  |  |
| **Therapeutic interventions** | Aspirin  Aspirin plus LMWH  G-CSF  Hydroxychloroquine  Intralipid  IVIG  Leukocyte immune therapy  LMWH  Prednisone plus progesterone plus aspirin  Progesterone | | |  |  |  |
| **Reference treatment** | Placebo | | |  |  |  |
| **Total studies:** 15  **Total participants:** 3525 | **Network estimate** | **Risk of bias** | **Indirectness** | **Inconsistency** | **Imprecision** | **Certainty** |
| **Aspirin versus placebo** | 0.97 (0.40 to 2.45) | Serious^1^ | Not serious | Not serious | Serious^2^ | **LOW**  Downgraded due to serious risk of bias and imprecision |
| **Aspirin plus LMWH versus placebo** | 1.02 (0.41 to 2.45) | Serious^1^ | Not serious | Not serious | Serious^2^ | **LOW**  Downgraded due to serious risk of bias and imprecision |
| **G-CSF versus placebo** | 0.63 (0.14 to 2.68) | Not serious | Not serious | Not serious | Serious^2^ | **MODERATE**  Downgraded due to serious imprecision |
| **Hydroxychloroquine versus placebo** | 1.24 (0.06 to 32.60) | Not serious | Not serious | Not serious |  | **MODERATE**  Downgraded due to serious imprecision |
| **Intralipid versus placebo** | 1.46 (0.43 to 5.11) | Not serious | Not serious | Not serious | Serious^2^ | **MODERATE**  Downgraded due to serious imprecision |
| **IVIG versus placebo** | 1.25 (0.52 to 3.11) | Not serious | Not serious | Not serious | Serious^2^ | **MODERATE**  Downgraded due to serious imprecision |
| **Leukocyte immune therapy versus placebo** | 0.72 (0.18 to 2.66) | Not serious | Not serious | Not serious | Serious^2^ | **MODERATE**  Downgraded due to serious imprecision |
| **LMWH versus placebo** | 0.70 (0.33 to 1.49) | Serious^1^ | Not serious | Not serious | Serious^2^ | **LOW**  Downgraded due to serious risk of bias and imprecision |
| **Prednisone plus progesterone plus aspirin versus placebo** | 3.90 (0.92 to 21.09) | Not serious | Not serious | Not serious | Serious^2^ | **MODERATE**  Downgraded due to serious imprecision |
| **Progesterone versus placebo** | 1.12 (0.46 to 2.76) | Not serious | Not serious | Not serious | Serious^2^ | **MODERATE**  Downgraded due to serious imprecision |
| **Table definitions**  *Solid lines represent direct comparison.  **Network estimates are reported as odds ratio with corresponding 95% credible intervals. Results are expressed as credible intervals as opposed to the confidence intervals since Bayesian analysis has been conducted, and these should be interpreted as the interval where there is a 95% probability that the values of the odds ratio will lie. | | | | | | |
| **GRADE Working Group Grades of Evidence (or certainty of the evidence)**  **High quality**: We are very confident the true effect lies close to that of the estimate of the effect.  **Moderate quality**: We are moderately confident in the effect estimate: The true effect is likely to be close to the estimate of effect, but there is a possibility that it is substantially different.  **Low quality**: Our confidence in the effect estimate is limited: The true effect may be substantially different from the estimate of effect.  **Very low quality**: We have very little confidence in the effect estimate: The true effect Is likely to be substantially different from the estimate of the effect. | | | | | | |
| **Explanatory footnotes**  **1:** Downgraded due to serious overall risk of bias across trials in the comparison in question.  **2:** Downgraded due to serious imprecision, namely with 95% credible intervals, which include widely disparate conclusions regarding the outcome in question. | | | | | | |

G-CSF, granulocyte colony-stimulating factor; IVIG, intravenous immunoglobulin G; LMWH, low-molecular-weight heparin.
