# Supplementary material for: Efficacy of therapeutic interventions for idiopathic recurrent pregnancy loss: a systematic review and network meta-analysis
Source: Front Med (Lausanne). 2025 May 14;12:1569819. doi: 10.3389/fmed.2025.1569819 (PMC12116322; doi:10.3389/fmed.2025.1569819)
Supplement: Supplementary file 12 [file Table_6.DOCX]

**Supplementary material**

**Supplementary Table S6.** Characteristics of trials including the outcome serious adverse events.

| **Study** | **Treatment** | **Responders** | **Sample Size** |
| --- | --- | --- | --- |
| Coomarasamy *et al.* (2015) | Progesterone | 3 | 404 |
| Coomarasamy *et al.* (2015) | Placebo | 2 | 432 |
| Eapen *et al.* (2019) | G-CSF | 4 | 76 |
| Eapen *et al.* (2019) | Placebo | 2 | 74 |
| Schleussner *et al.* (2015) | Vitamins containing folic acid and LMWH | 41 | 232 |
| Schleussner *et al.* (2015) | Multivitamins containing folic acid | 26 | 217 |

G-CSF, granulocyte colony-stimulating factor. LMWH, low-molecular-weight heparin.
