# Supplementary material for: Efficacy of therapeutic interventions for idiopathic recurrent pregnancy loss: a systematic review and network meta-analysis
Source: Front Med (Lausanne). 2025 May 14;12:1569819. doi: 10.3389/fmed.2025.1569819 (PMC12116322; doi:10.3389/fmed.2025.1569819)
Supplement: Supplementary file 13 [file Table_7.DOCX]

**Supplementary material**

**Supplementary Table S7.** Characteristics of trials including the outcome adverse events.

| **Study** | **Treatment** | **Responders** | **Sample size** |
| --- | --- | --- | --- |
| Akbari *et al.(*2022) | Aspirin plus LMWH | 3 | 85 |
| Blomqvist *et al.* (2018) | Aspirin | 34 | 200 |
| Blomqvist *et al*. (2018) | Placebo | 21 | 200 |
| Dolitzky *et al.* (2006) | LMWH | 0 | 54 |
| Dolitzky *et al.* (2006) | Aspirin | 0 | 50 |
| Eapen *et al.* (2019) | G-CSF | 52 | 76 |
| Eapen *et al.* (2019) | Placebo | 43 | 74 |
| El-Zibdeh (2005) | Dydrogesterone | 0 | 82 |
| El-Zibdeh (2005) | hCG | 0 | 50 |
| El-Zibdeh (2005) | Placebo | 0 | 48 |
| Meng *et al.* (2016) | Intralipid | 0 | 96 |
| Meng *et al.* (2016) | IVIG | 5 | 96 |
| Rehder (1994) | IVIG | 1 | 33 |
| Rehder (1994) | Placebo | 5 | 31 |
| Scarpellini *et al.* (2009) | G-CSF | 3 | 35 |
| Scarpellini *et al.* (2009) | Placebo | 1 | 33 |
| Schleussner *et al.* (2015) | Vitamins containing folic acid and LMWH | 41 | 232 |
| Schleussner *et al.* (2015) | Multivitamins containing folic acid | 43 | 217 |
| Shaaban *et al.* (2017) | Folic acid plus LMWH | 32 | 150 |
| Xu *et al.* (2018) | LMWH | 12 | 60 |
| Xu *et al.* (2018) | Progesterone plus hCG | 14 | 60 |
| Yamada *et al.* (2022) | IVIG | 23 | 50 |
| Yamada *et al.* (2022) | Placebo | 3 | 49 |

G-CSF, granulocyte colony-stimulating factor; hCG, human chorionic gonadotropin; IVIG, intravenous immunoglobulin G; LMWH, low-molecular-weight heparin.
