# Supplementary material for: Efficacy of therapeutic interventions for idiopathic recurrent pregnancy loss: a systematic review and network meta-analysis
Source: Front Med (Lausanne). 2025 May 14;12:1569819. doi: 10.3389/fmed.2025.1569819 (PMC12116322; doi:10.3389/fmed.2025.1569819)
Supplement: Supplementary file 14 [file Table_8.DOCX]

**Supplementary material**

**Supplementary Table S8.** Network meta-analysis models for the outcome live birth rate.

| Parameters | FE model | RE model | UME model |
| --- | --- | --- | --- |
| Data points | 58 | 58 | 58 |
| Dbar | 106.55 | 58.08 | 57.26 |
| pD | 40.48 | 51.66 | 52.75 |
| DIC | 147.02 | 109.74 | 110.01 |
| Tau | - | 0.6 | 0.63 |
| SD | - | 0.59 | 0.61 |
| SD 95% CrI LB | - | 0.36 | 0.35 |
| SD 95% CrI UB | - | 0.93 | 1.04 |

CrI, credible interval; Dbar, mean sum of residual deviance; DIC, Deviance Information Criterion; FE, fixed-effects; LB, lower bound; pD, sum of leverage, also known as the effective number of parameters; RE, random-effects; SD, standard deviation; UB, upper bound; UME, unrelated mean effects.
