# Supplementary material for: Efficacy of therapeutic interventions for idiopathic recurrent pregnancy loss: a systematic review and network meta-analysis
Source: Front Med (Lausanne). 2025 May 14;12:1569819. doi: 10.3389/fmed.2025.1569819 (PMC12116322; doi:10.3389/fmed.2025.1569819)
Supplement: Supplementary file 15 [file Table_9.DOCX]

**Supplementary material**

**Supplementary Table S9.** Network meta-analysis models for the outcome miscarriage rate.

| Parameters | FE model | RE model | UME model |
| --- | --- | --- | --- |
| Data points | 69 | 69 | 69 |
| Dbar | 123.81 | 68.7 | 68.16 |
| pD | 48.84 | 61.48 | 63.59 |
| DIC | 172.65 | 130.17 | 131.76 |
| Tau | - | 0.61 | 0.67 |
| SD | - | 0.6 | 0.65 |
| SD95%CrILB | - | 0.38 | 0.39 |
| SD95%CrIUB | - | 0.91 | 1.06 |

CrI, credible interval; Dbar, mean sum of residual deviance; DIC, Deviance Information Criterion; FE, fixed-effects; LB, lower bound; pD, sum of leverage, also known as the effective number of parameters; RE, random-effects; SD, standard deviation; UB, upper bound; UME, unrelated mean effects.
