# Supplementary material for: Efficacy of therapeutic interventions for idiopathic recurrent pregnancy loss: a systematic review and network meta-analysis
Source: Front Med (Lausanne). 2025 May 14;12:1569819. doi: 10.3389/fmed.2025.1569819 (PMC12116322; doi:10.3389/fmed.2025.1569819)
Supplement: Supplementary file 16 [file Table_10.DOCX]

**Supplementary material**

**Supplementary Table S10.** Network meta-analysis models for the outcome trial discontinuation.

| Parameters | FE model | RE model | UME model |
| --- | --- | --- | --- |
| Data points | 32 | 32 | 32 |
| Dbar | 38.45 | 37.18 | 36.16 |
| pD | 25.83 | 25.65 | 27.1 |
| DIC | 64.28 | 62.84 | 63.26 |
| Tau | - | 0.24 | 0.61 |
| SD | - | 0.18 | 0.45 |
| SD95%CrILB | - | 0.03 | 0.02 |
| SD95%CrIUB | - | 0.76 | 2.1 |

CrI, credible interval; Dbar, mean sum of residual deviance; DIC, Deviance Information Criterion; FE, fixed-effects; LB, lower bound; pD, sum of leverage, also known as the effective number of parameters; RE, random-effects; SD, standard deviation; UB, upper bound; UME, unrelated mean effects.
